# Integrating Transcriptional, Metabolic, and Physiological Responses to Drought Stress in *Ilex paraguariensis* Roots

**DOI:** 10.3390/plants12132404

**Published:** 2023-06-21

**Authors:** Edgardo H. Avico, Raúl M. Acevedo, María J. Duarte, Acácio Rodrigues Salvador, Adriano Nunes-Nesi, Oscar A. Ruiz, Pedro A. Sansberro

**Affiliations:** 1 Laboratorio de Biotecnología Aplicada y Genómica Funcional, Instituto de Botánica del Nordeste (IBONE-CONICET), Facultad de Ciencias Agrarias, Universidad Nacional del Nordeste, Sgto. Cabral 2131, Corrientes W3402BKG, Argentina; eavico88@gmail.com (E.H.A.); rm.acevedo@agr.unne.edu.ar (R.M.A.); mj.duarte@conicet.gov.ar (M.J.D.); 2 National Institute of Science and Technology on Plant Physiology under Stress Conditions, Departamento de Biologia Vegetal, Universidade Federal de Viçosa, Viçosa 36570-900, MG, Brazil; acacio.fvufv@gmail.com (A.R.S.); nunesnesi@gmail.com (A.N.-N.); 3 Unidad de Biotecnología 1, IIB-INTECH (UNSAM-CONICET), Chascomús B7130IWA, Argentina; ruiz@intech.gov.ar

**Keywords:** *Ilex paraguariensis*, drought stress response, root transcriptome, RNA-seq

## Abstract

The appearance of water stress episodes triggers leaf abscission and decreases *Ilex paraguariensis* yield. To explore the mechanisms that allow it to overcome dehydration, we investigated how the root gene expression varied between water-stressed and non-stressed plants and how the modulation of gene expression was linked to metabolite composition and physiological status. After water deprivation, 5160 differentially expressed transcripts were obtained through RNA-seq. The functional enrichment of induced transcripts revealed significant transcriptional remodelling of stress-related perception, signalling, transcription, and metabolism. Simultaneously, the induction of the enzyme 9-cis-expoxycarotenoid dioxygenase (NCED) transcripts reflected the central role of the hormone abscisic acid in this response. Consequently, the total content of amino acids and soluble sugars increased, and that of starch decreased. Likewise, osmotic adjustment and radical growth were significantly promoted to preserve cell membranes and water uptake. This study provides a valuable resource for future research to understand the molecular adaptation of *I. paraguariensis* plants under drought conditions and facilitates the exploration of drought-tolerant candidate genes.

## 1. Introduction

*Ilex paraguariensis* St. Hil. (Aquifoliaceae) is an evergreen tree naturally distributed in South America between 22° and 30° south latitude and between 48° and 56° west longitude, with altitudes between 400 and 1800 m above sea level and annual rainfall between 1100 and 2300 mm [1]. It is extensively cultivated as monoculture in South Brazil, Northeast Argentine, and West Paraguay to prepare a tea-like infusion with several pharmacological properties [2]. However, due to climate change, extensive periods of drought and extreme temperatures are frequent [3] and trigger leaf abscission, constraining the agronomical yields and survival of established plantations.

Under these circumstances, depending on the magnitude of the strain and the stress duration, *I. paraguariensis* can activate several morphological, physiological, and biochemical adaptation mechanisms to cope with environmental stresses. For example, our previous studies showed that a drought-tolerant cultivar responds early to soil water shortage by promoting stomatal closure [4]. However, when the water deficit becomes severe, *I. paraguariensis* displays other acclimation mechanisms, including root stimulation and further changes in the leaves, such as osmotic adjustment, photoprotection of the photosynthetic apparatus, and regulation of non-structural carbohydrates and amino acid metabolism [5]. Although root growth is stimulated by stress, little is known about how the root system contributes to *I. paraguariensis* acclimation.

Usually, tree roots can respond to drought through various strategies that enable them to avoid and tolerate water scarcity. Responses include root biomass adjustments, anatomical alterations, and physiological acclimations [6]. The molecular mechanisms underlying these responses are somewhat characterised and involve stress signalling and the induction of numerous genes, leading to the activation of tolerance pathways [7]. In addition, mycorrhizas seem to play a protective role [8].

The root system architecture has been the target of modern breeding programs to develop drought-tolerant varieties. The overall root system size is considered one of the main traits that maintains plant productivity, since it is related to acquiring water and nutrients from the soil and should be accompanied by a balanced leaf surface area ratio [9]. The root system components underlying drought tolerance responses include root diameter, root tissue density, specific root length, and the presence of young roots with abundant root hairs to enlarge the contact surface between soil and roots [10]. 

The development of a root system that ensures an adequate supply of water in restrictive conditions is controlled by a wide range of molecular mechanisms, including the signalling of the soil water status toward the shoot and changes in the regulation of gene expression that subsequently control the molecular pathways of the plant’s drought response [11]. Understanding this molecular network, its components, and its regulatory mechanisms is essential to designing genotypes with enhanced tolerance to water deficits.

Here, we investigated (i) how the root gene expression varies under water scarcity concerning well-watered conditions, (ii) in what ways this variation is linked to the root metabolite composition and physiological status, and finally, (iii) an integrative analysis of transcriptional, metabolomic, and physiological responses of the whole plant. *Ilex paraguariensis* modifies its morphology to ensure the provision of resources in limiting situations. Intricate mechanisms control such plasticity, including perception, signal integration, and stress response. In the aerial part of the plant, this is related to optimising light uptake and acclimation, while root growth is promoted to ensure water and nutrient extraction. In addition, an efficient osmotic adjustment between root and leaves guarantees the water flux through the soil–atmosphere continuum.

## 2. Results

### 2.1. Root Physiological and Morphological Changes in Response to Drought

The effect of water shortage on the root relative water content and osmotic potential was analysed. The root relative water content decreased from 89.2 ± 3.2% (soil at field capacity) to 83.3 ± 2.2% (*p* < 0.05) and 54.5 ± 2.7% (*p* < 0.0001) when the soil water potential dropped to −1 (moderate stress) and −2 MPa (severe stress). Furthermore, this variation was correlated with a pronounced decrease in the root osmotic potential through an active solute accumulation. Consequently, a significant osmotic adjustment concerning the well-watered conditions was evident under moderate (−0.47 ± 0.14 MPa, *p* < 0.05) and severe (−0.83± 0.04 MPa, *p* < 0.01) stress.

**Figure 1:**
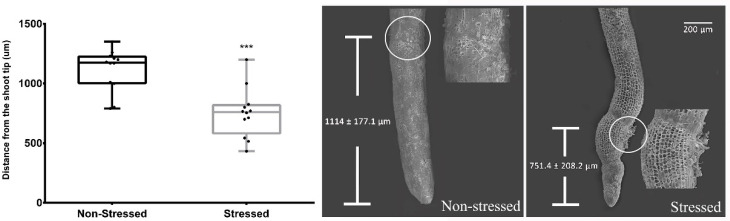
Effect of severe drought stress (Ψ_soil_ = −2 MPa) on root hair formation. **Left**: distance of root hair zone formation from the root tip. Bars indicate mean ± SE (*n* = 10); three asterisks denote *p* < 0.001 for the *t*-test. **Right**: scanning electron microscopy images of the root tips from non-stressed and stressed roots. Bar indicates 200 µm.

Besides physiological adjustments, *I. paraguariensis* plants also undergo a morphological adaptation to drought. For example, stressed plants modified their growth pattern to recover available water by prioritising root growth. As a result, the variation in root dry weight during the 22-day experiment ranged from 1.84 ± 0.12 to 5.07 ± 0.7 g per plant (*p* < 0.01) when the soil water potential reached −2 MPa. In addition, by scanning electron microscopy, we also verified a variation in the origin zone of root hair formation that contributes to water absorption. In this sense, using shoot tips collected from primary roots of similar diameter, it was observed that the distance from the root tip to the first root hair (Figure 1) was significantly lower in stressed roots (751.4 ± 60.1 μm) compared to those that grew in well-watered conditions (1114 ± 51.1 μm).

### 2.2. Transcriptome Sequencing, Assembly and Differential Expression Analysis

High-quality root RNA was isolated from three drought-stressed (Ψ_soil_ = −2 MPa) and three non-stressed plants to construct six cDNA libraries and was sequenced on an Illumina HiSeq 1500 platform. A total of 312,265,792 paired-end reads (2 × 100 bp) were produced, with a 44% GC average content. After discarding the low-quality reads, a de novo transcriptome was assembled with 231,263,325 bases (40.92% GC) contained in 190,474 transcript contigs with an N50 length of 945 bp. Sixty-two transcripts previously obtained by the Sanger method were employed to validate the nucleotide accuracy of the assembled sequences in the de novo transcriptome using BLASTN (Supplementary Table S1). These sequences were previously obtained from the same genotype subjected to a similar drought stress experiment [4,11]. All Sanger sequences were present in the transcriptome, and among them, fifty-six sequences presented nucleotide identities ranging from 98% to 100% along the aligned fragments. 

As a result of the RNAseq bioinformatics data analysis, 1389 transcripts were up-regulated, and 2983 were down-regulated (expression change |FC| ≥ 4 and FDR < 0.001) compared to non-stressed roots (Supplementary Figure S1). Then, nine transcripts were randomly selected to quantify their expression variation using real-time qPCR (Supplementary Table S2), corroborating with that calculated from the RNA-seq data.

The expression values obtained through both techniques were consistent (R^2^ = 0.934). This equivalence between RNA-seq and RT-qPCR supports an overall validity of drought-expressed transcripts (DETs) determined by bioinformatic methods from RNA-seq data (Supplementary Figure S2). In this context, the *GBE1* (*1,4-alpha-glucan branching enzyme*), *GolS* (*galactinol synthase*), *ACC oxidase* (*aminocyclopropanecarboxylate oxidase*), *TreS* (*trehalose synthase*), and *AOX* (*alternative oxidase*) transcripts increased their expression, while *PPO* (*polyphenol oxidase*), *RafS* (*raffinose synthase*), and *RSI-1* (*RS-1 protein*) decreased their expression in response to water deficit. The uncharacterized transcript c58016_g2_i2 maintained its expression levels without changes.

### 2.3. Transcriptome Functional Annotation and Metabolic Pathways Analysis

Using BLASTx, 63.3% of the assembled transcripts were annotated by similarity with accessions from the TrEMBL protein sequence database and 41.9% with ortholog sequences stored in the Swiss-Prot database. Furthermore, 39.7% of the transcriptome was annotated with at least one GO term and 20.9% with a sequence from the EggNOG database. A signal peptide was identified in 2.2% of the transcripts; 0.1% was annotated as ribosomal RNA residual contamination.

Functional enrichment using Fisher’s exact test allowed the delimiting of 84 functions from the up-regulated genes (Supplementary Table S3), while 288 functions were enriched from those transcripts that decreased their expression in response to drought (Supplementary Table S4). Table 1 specifies the number of GO domains enriched by the DETs.

Within the biological process GO domain, subcategories related to cellulose catabolism, viral RNA replication, signalling pathways, and hormonal interaction were enriched by up-regulated genes (Supplementary Figure S3). Moreover, on the other hand, down-regulated genes were responsible for the decline of subcategories belonging to the synthesis of the secondary cell wall, cellulose, and lignin; the catabolic process of pectins, lipids, and hydrogen peroxide; and defense against biotic (fungi and bacteria) and abiotic (water deficit and cold) stress (Supplementary Figure S4).

Regarding the cellular component GO category, the functions overrepresented by the induced genes correspond mainly to the nucleus subcategory, followed by the extracellular region (Supplementary Figure S5). Functions repressed by genes with decreased expression mainly include integral components of the membrane and structures such as apoplasts, phragmoplasts, and plasmodesmata (Supplementary Figure S6).

Finally, concerning the molecular function category, the most enriched subcategories correspond to molecules that contribute to the structural integrity of ribosomes, RNA-dependent transcriptase-type and cellulose degradation enzymes (Supplementary Figure S7). The most frequent GO terms in genes with decreased expression are those involved in the regulatory aspect, corresponding to the modulation of the transcription subcategory by binding to a specific genomic DNA sequence. Terms in this subcategory related to metabolic pathways include cellulose synthase activity, pectin esterase activity, and nutrient storage (Supplementary Figure S8).

#### 2.3.1. Hormone Signalling

**Figure 2:**
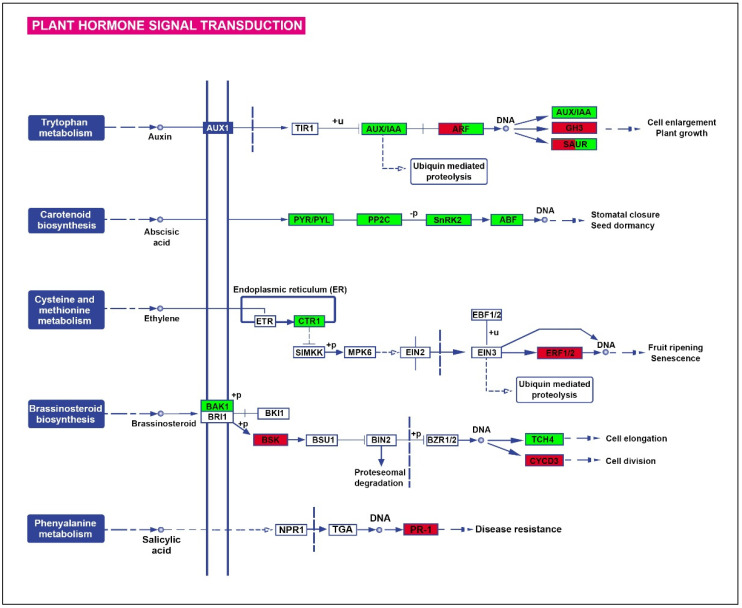
KEGG diagram of plant hormone signal transduction in *Ilex paraguariensis* root subjected to a severe drought stress (Ψ_soil_ = −2 MPa) episode. Green and red indicate increased and decreased expression (FC ≥ 2, FDR < 0.001).

Transcripts involved in signal transduction mediated by abscisic acid (ABA), ethylene, auxins, cytokinins, jasmonic acid, brassinosteroids, and salicylic acid were identified. Results indicated an increase in ABA signalling gene expression and a general decrease in the transcription of genes related to other hormones involved in different developmental processes. In this context, the KEGG map (Figure 2) highlights the increased transcription of genes involved in ABA-mediated signalling processes such as PYR/PYL (T1), PP2C (T2, T3), SnRK2 (T4), and ABF (T5) (Supplementary Table S5). Likewise, a repression in the carotenoid cleavage dioxygenase 8 (CCD8, T6) expression and an accumulation of mRNAs encoding the NCED (9-cis-epoxycarotenoid dioxygenases, T7) enzyme were observed. Consequently, ABA3 (T8) decreased, activating the ABA aldehyde oxidase (AAO) expression and converting ABA-aldehyde to ABA. It is worth noting that an increase in the expression of transcripts involved in putrescine biosynthesis from L-ornithine was identified. Thus, the expression of transcripts encoding glutamate N-acetyltransferase (T9) and ornithine decarboxylase (T10) increased by fourteen- and six-fold to produce putrescine.

Drought also stimulated the expression of transcripts encoding ACC oxidase (T11), which catalyses the last step in ethylene formation. In addition, positive variation was observed in the CTR1 (T12) transcript, which is considered a negative regulator of ethylene response. Consequently, the salicylic acid biosynthesis was probably altered by the upregulation of MES1 (T13) induced by ethylene.

Finally, further variation in the expression of transcripts associated with auxin metabolism (SAUR32, T14), signalling (IAA10, T15), and inactivation (GH3, T16) was noticed. Likewise, the expression of several transcripts related to cytokinins (T17, T18, T19), gibberellins (T20, T21, T22, T23), jasmonates (T24, T25, T26), and brassinosteroids (T27, T28, T29, T30, T31) decreased under stress (Supplementary Table S5).

Among the mRNAs encoding other proteins involved in integrating internal and external stimuli to the cell, the overexpression of transcripts encoding the TOR (T32) protein is worth highlighting.

#### 2.3.2. Transcription Factors

One hundred sixty-three up-regulated and 927 down-regulated transcript factors (TFs) were identified using the PlantTFDB v4.0 database. NAC, bHLH, ERF, related to MYB, B3, C2H2, MYB, WRKY, bZIP, and C3H were the most abundant TF families (Supplementary Figure S9). An increase in NAC, ERF, MYB, and bZIP families was observed in response to drought.

An interaction network of stress-stimulated transcription factors was constructed (Supplementary Figure S10) using the online tool STRING 10.0 (https://string-db.org/) and the *Arabidopsis thaliana* database hosted on the site. Sixty-six TFs were functionally annotated, identifying four main interaction clusters organised around HSFC1 (T33), ATHB-12 (T34)/ATBH-7 (T35)/RD26 (T36), SIGE (T37), and HY5 (T38). Colour-coded transcription factors that were assigned to various GO biological processes using STRING algorithms include responses to ethylene: MYB59 (T39), MYB73 (T40), ERF10 (T41), EBP (T42), ARR2 (T43), WRKY4 (T44); ABA responses: RD26 (T36), DREB2C (T45), ATHB-12 (T34), ATHB-7 (T35), GBF3 (T46), HY5 (T38), ARF2 (T47), MYB73 (T40); auxin responses: IAA6 (T48), IAA29 (T49), HB2 (T50), ARF2 (T47), RVE1 (T51), HB40 (T52); and stress responses: WRKY33 (T53), ATHB-12 (T34), ATHB-7 (T35), SIGE (T37), and ATAF1 (T54). In addition, TFs involved in the regulation of root development, such as the *A. thaliana* response-regulating proteins ARR1 (T55), ARR2 (T43), ARR11 (T56), and ZAT11 (T57), are highlighted.

#### 2.3.3. Carbohydrates

An active transcription profile variation in the starch and sucrose metabolism was observed (Supplementary Figure S11). In response to dehydration, transcripts encoding the 1,4-alpha-glucan branching enzyme (T58), involved in starch synthesis, as well as those that participate in its hydrolysis, including alpha-amylase (T59) and isoamylase (T60), increased their expression. Likewise, the differential expression of several transcripts involved in sucrose biosynthesis (sucrose synthase, T61–T65) and hydrolysis (beta-fructofuranosidase, T66) was observed. Likewise, the expression of transcripts encoding trehalose 6-phosphate phosphatase/synthetase (T67/T68) to form trehalose from UDP-glucose and D-glucose-6P was strongly stimulated. Concurrently, the expression of several transcripts encoding enzymes that catalyse the production of cellobiose (T69, T70, T71) was repressed in the stressed roots. 

The interconversion of UDP-glucose and UDP-galactose by UDP-glucose 4-epimerase (GalE, T72), followed by the conversion of galactose to galactinol, by galactinol synthase (GolS, T73) (Supplementary Figure S12), was stimulated at the transcriptional level. However, the expression of transcripts encoding raffinose synthase (T74) to produce raffinose was strongly repressed (FC = −23). In contrast, the alpha-galactosidase (T75) gene expression to produce galactose was promoted in response to dehydration. 

Most transcripts encoding glycolytic enzymes were repressed under severe water stress, including the regulatory proteins glucokinase (T76), involved in the entry of glucose into the glycolysis pathway, 6-phosphofructokinase 1 (T77), responsible for producing fructose 1,6-bisphosphate, and pyruvate kinase (T78 and T79), which catalyses the last step to obtain pyruvate (Supplementary Figure S13). In addition, various pyruvate dehydrogenase complex components, including pyruvate dehydrogenase component E1 (T80), pyruvate decarboxylase (T81), pyruvate dehydrogenase component E2 (T82), and dihydrolipoamide dehydrogenase (T83) were down-regulated during stress.

#### 2.3.4. Amino Acids and Proteins

In *I. paraguariensis* roots, drought promoted the biosynthesis of valine, leucine, and isoleucine from pyruvate (Supplementary Figure S14). Thus, an increase in ketoacid reductoisomerase (ilvC, T84), acetolactate synth I/III small subunit (ilvH/ilvN, T85), and 3-isopropyl malate large subunit/(R)-2-methyl maleate dehydratase leuC (T86)-related genes, was observed.

Concurrently, further variation in the polypeptide and protein profile was confirmed, comprising the overexpression of numerous transcripts encoding enzymes with proteolytic activity, including aspartyl-proteases (T87), cysteine-proteases (T88, T89, T90), ubiquitin-carboxy-terminal-hydrolases (T89), metalloproteases (T91, T92, T93, T94, T95), serine-peptidases (T96, T97, T98), and threonine-peptidases (T90 and T100). Similarly, an increase in the transcription of several mRNAs related to ribosome assembly was detected, including proteins such as ERAL1 (ribosomal genesis factor, T101), MRPL46 (large subunit ribosomal protein L46, T102), and RP-S25e (small subunit ribosomal protein, T103); as well as those associated with pre-translational mRNA transport, processing, and stability, including UTP5 19 and 25 (small nucleolar RNA-associated proteins 19 and 25 U3, T104, and T105, respectively), adenylate kinase (T106), transportin 1 (T107), and factors involved in translation such as EIF1, EIF4A, EIF5A (translation initiation factors 1, 4A and 5A, T108-T110), and ERF3 (subunit 3 of the peptide chain releasing factor, T111). Consequently, the overexpression of transcripts encoding aspartyl-tRNA synthetase (T112) and valyl-tRNA synthetase (T113), related to the protein synthesis process, was observed. Lastly, the transcription of genes encoding the LEA proteins (late embryogenesis abundant proteins, T114–T117) was stimulated in the stressed roots.

**Figure 3:**
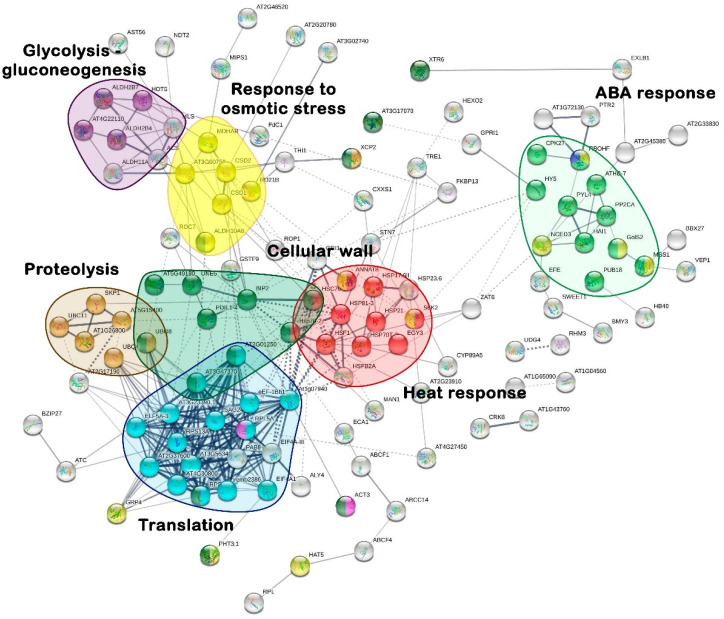
Interaction network of predicted proteins from up-regulated genes. The analysis of functional associations was performed with the *A. thaliana* database based on information from root RNA-seq data of drought-stressed *I. paraguariensis* plants. The line width indicates the type and reliability of evidence about the interaction. A confidence value of 0.4 was used. The GO biological process to which the proteins (nodes) of the identified clusters belong is indicated with a key colour: response to osmotic stress (yellow), translation (light blue), response to abscisic acid (light green), proteolysis (brown), response to heat (red), glycolysis/gluconeogenesis (purple), and cell wall (dark green).

After that, protein interactions were analysed using the STRING tool. First, the 1367 overexpressed transcripts were aligned against the *A. thaliana* database, and 199 were annotated with the identity of at least one protein. Next, an interaction network was generated where the line width and colour intensity represented their interaction level and determined their closeness (Figure 3).

The cellular root interactome showed six well-defined clusters, where the most compact with the highest protein number was associated with the GO biological process “translation”, consisting of 18 proteins (light blue). The small 6-protein “proteolysis” cluster closely interacted with the “translation” one. Similarly, two nearby groups belonging to the GO categories “cellular wall” and “heat responses”, with ten annotated proteins, were identified. In addition, three sets of proteins with co-expressed transcripts defining the interaction of “ABA response” (green), “osmotic stress response” (yellow), and “glycolysis/glycogenolysis” (purple) clusters were apparent, containing 10, 6, and 4 annotated proteins, respectively.

Concerning the protein identity assignment, the EF1Alpha protein (At1g69410.1, T118) was differentiated in the “translation” category as an interconnection node with the “heat response”. Likewise, the proteins ELF5A-3 (At1g69410.1, T119), SAG24 (At1g66580.1, T120), RPS18C (T121), and other ribosomal structural proteins were identified. Linked to “proteolysis”, nodes related to polypeptide ubiquitination and proteasome degradation were confirmed, including the polyubiquitin 4 and 8 proteins (At5g20620.1 and At3g09790.1, T122 and T123, respectively), UBC11 (At3g08690.2, T124), and SKP1 (At1g75950.1, T125). Regarding the “cell wall” cluster, transcripts corresponding to the pectin methylesterase 58 (At5g49180.1, T126), actin (At3g53750.1, T127), xylematic cysteine peptidase 2 (At1g20850.1, T128), XTR6 (At4g25810.1, T129), peroxidases (At3g17070.1, T130), and AIR1 (At2g04160.1, T131) proteins were observed. Among the “heat response” proteins, HSP81-3 (At5g56010.1, T132), HSP70 (At5g02500.1, T133), ANNAT8 (At5g12380.1, T134), and EGY3 (At1g17870.1, T135) were annotated. For the “ABA response”, PP2CA (T2/T3), PYL4 (T1), PUB18 (At1g10560.1, T136), GolS2 (At1g56600.1, T137), and CPK2 (At4g04700.1, T138) proteins were defined. Among the “osmotic stress response”, MDHAR (At3g09940.1, T139), CSD2 (AT2G28190.1, T140), RD21B (At5g43060.1, T141), ALDH10A (At1g74920.1, T142), MSS1 (At5g26340.1, T143), NCED3 (T7), GRP4 (At3g23830.2, T144), RCD1 (At1g32230.1, T145), and RBOHF (At1g64060.1, T146) proteins were identified. Finally, for the “glycolysis/gluconeogenesis” category, a small cluster of four protein nodes was identified, including the alcohol dehydrogenases similar to GroES, two isoforms of AT4G22110 (T147 and T148), HOT5 (At5g43940.2, T149), and ALDH2B4 (At3g48000.1, T150).

### 2.4. Metabolites Variation

Through the combination of different analytical tools, 55 compounds were quantified, including amino acids (20), organic acids or their anions (13), carbohydrates (13), amines (2), nucleobases (2), polyol (1), and others (4). 

**Figure 4:**
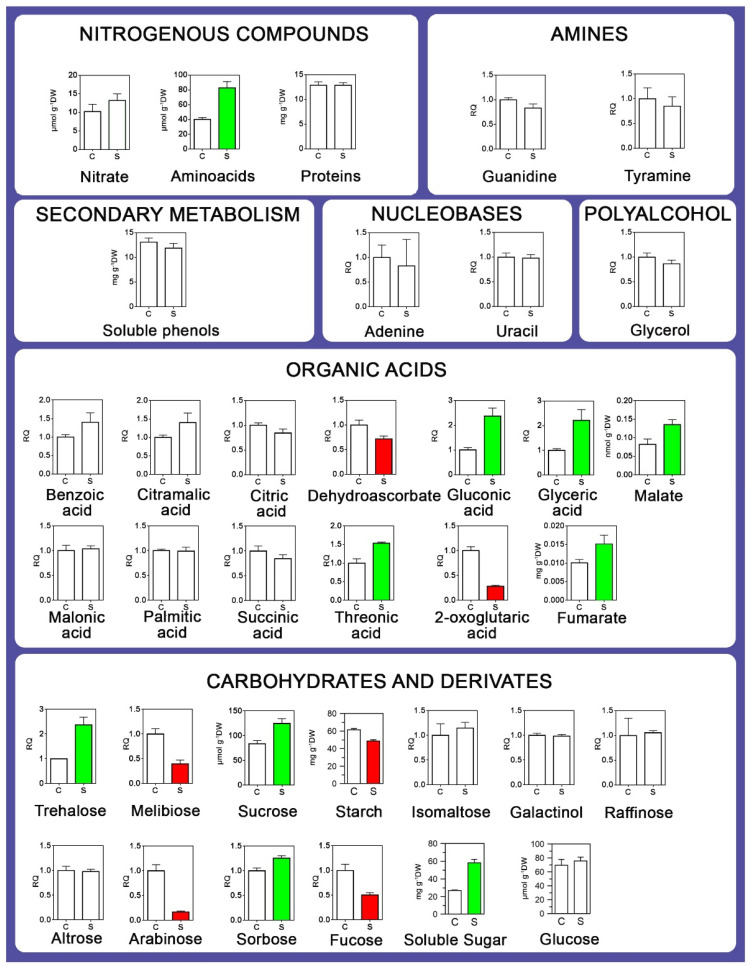
Metabolomic profile of *I. paraguariensis* roots subjected to a severe drought stress (Ψ_soil_ = −2 MPa) episode. Bars represent the mean of three biological replicates ± SD. Values are expressed in absolute or relative terms. Bars with different colours indicate variation in the content of biological compounds between treatments (*t*-test, *p* < 0.05). Red and green indicate lower and higher levels than the well-watered control. The white colour indicates no variation. C, non-stressed (Ψ_soil_ ~ −0.04 Mpa); S, stressed (Ψ_soil_ = −2 Mpa) plants. RQ, relative quantity.

Principal component analysis indicated differences in the metabolic profiles of roots between control and stressed plants. CP1 explained 44.24% of the total variation, allowing the separation of controls and stressed treatments, while the CP2 component explained 24.89%. The root metabolite profile of the well-watered plants was primarily correlated with those metabolites that decreased their content under stress. In contrast, the stressed root metabolic profile was mainly associated with increased metabolite levels (Supplementary Figure S15). For example, the total amino acid content and 17 other compounds, including carbohydrates, organic acids, and amino acids, increased due to water deprivation, while eight metabolites belonging to the same categories diminished their concentration (Figure 4). The total protein and nitrate content remained unchanged. 

**Figure 5:**
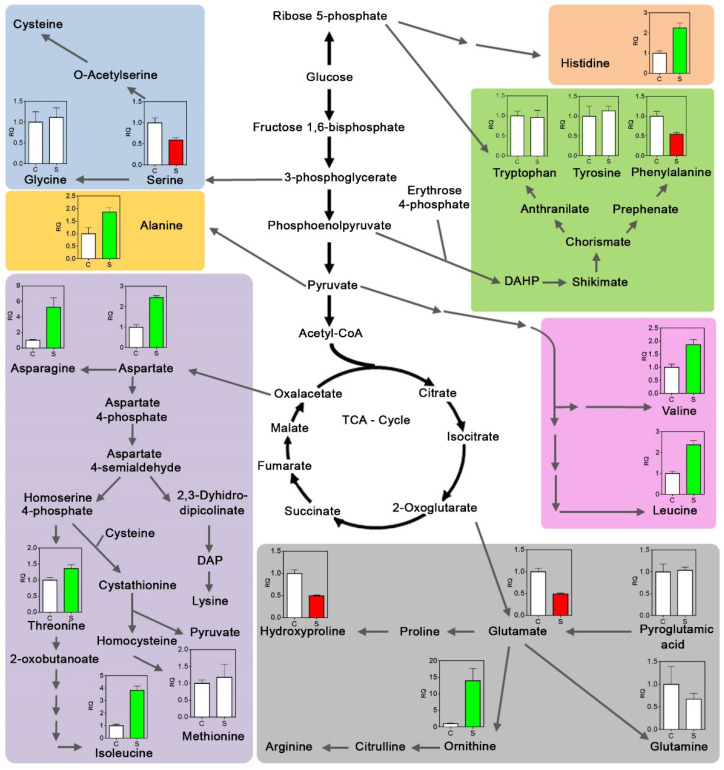
Schematic diagram of drought-induced changes in the *I. paraguariensis* root amino acid metabolism. Bars represent the mean of three biological replicates ± SD. Values are expressed in relative terms to control plant level. Bars with different colours indicate variation in the content of biological compounds between treatments (*t*-test, *p* < 0.05). Red and green indicate lower and higher levels than the well-watered control. White colour shows a non-significant difference concerning the well-watered conditions. C, non-stressed (Ψ_soil_ ~ −0.04 MPa); S, stressed (Ψ_soil_ = −2 MPa) plants. RQ, relative quantity.

Interestingly, due to dehydration, the total amino acid content in roots increased by two-fold. Among them, the basic amino acids asparagine and ornithine increased their concentration more than 5 and 13 times, respectively (Figure 5). Likewise, the branched-chain amino acids (leucine, isoleucine, and valine) contributed to the rise of total amino acids, duplicating or quadruplicating their contents. On the other hand, glycine, methionine, tryptophan, tyrosine, and the amino acid derivative pyroglutamic acid did not present significant variations. In contrast, the levels of glutamic acid, phenylalanine, hydroxyproline, and serine decreased by about half concerning the control treatment. Proline was not detected.

In response to the water shortage, the malate, glyceric acid, and gluconic acid contents increased, while the dehydroascorbate anion and 2-oxoglutaric acid dropped. On the other hand, no changes in the concentration of citric, malonic, palmitic, and succinic acids were observed (Figure 4). 

Finally, the starch content in the stressed roots decreased significantly from 61.4 ± 1.6 to 48.8 ± 1.4 mg·g^−1^ dw, and the soluble sugar content increased from 26.9 ± 0.7 to 58.2 ± 3.9 mg·g^−1^ dw (Figure 4), probably as a result of the starch and sucrose hydrolysis. The trehalose concentration increased by 236-fold. Arabinose (84%), melibiose (61%), and fucose (50%) decreased by 84, 61, and 50%. No variations in the altrose, isomaltose, galactinol, and raffinose content were detected.

## 3. Discussion

### 3.1. Root Phenotypic Plasticity in Response to Drought

Phenotypic plasticity is the ability of an organism to alter its phenotype in response to environmental variation. It may involve changes in physiology, morphology, anatomy, development, or resource allocation resulting from a complex synergistic developmental system controlled by many genes, gene products, and the environment [12]. Root phenotypic plasticity is vital in soil resource capture, especially in suboptimal water and nutrient availability environments. Root anatomical and architectural patterns determine the temporal and spatial distribution of root foraging in specific soil domains and, hence, the capture of mobile and immobile resources [13]. *Ilex paraguariensis* responds early to drought by displaying typical isohydric behaviour based on the strict regulation of stomatal conductance modulated by rapid changes in the hydraulic conductivity of the soil and roots [5]. As the stress becomes severe (Ψ_soil_ = −2 MPa), it displays a second defense mechanism based on increased compatible osmolytes in the leaves and roots, favouring an adequate osmotic adjustment and preserving the water moving through the soil-plant-atmosphere continuum, even when the leaf relative water content falls below 65% [5]. These resilience mechanisms allow them to preserve the functionality of cell membranes and protect the photosynthetic apparatus, normalising the CO_2_ assimilation rate 48 h after rehydration [4]. 

In addition, the volume of soil explored by the roots and the root surface area that interacts with the soil constitute the main parameters that determine the efficiency of a root system [14]. In the present work, we verified that, in response to severe water deficit, the cultivar under study increased almost three-times its root system for the same soil volume. Thus, the induced root growth is expected to respond to both parameters under field conditions. Additionally, root hair formation dramatically increases the area in which the roots interact with the soil and contributes to the acquisition of immobile nutrients [12]. Using scanning electron microscopy, we observed a variation in the root hair zone differentiation of stressed roots, placing them closer to the root apex (Figure 1). These results agree with those reported by Labdelli et al. [15]. Decreased soil water availability promotes significant changes in the piliferous layer, presuming an early maturation of the cortical cells, emitting root hairs to facilitate root penetration through mucilage exudation and rhizospheric microorganism interaction [14]. Although, based on simple descriptive observation, this finding circumscribes a reduction in the cell elongation zone and opens the doors to future studies to clarify the biochemical and histological mechanisms involved in the variation of *I. paraguariensis* root structure induced by water shortage.

### 3.2. Gene Identification Related to Hydrotropic Responses

#### 3.2.1. Hormonal Interaction and Signalling

The perception and signalling of water deficit to determine the adaptive root response constitute a complex process, where ABA plays a leading role as a system moderator [16]. For example, in *Arabidopsis thaliana*, ABA induces the expression of genes encoding auxin transporters, such as PIN2 and AUX1, which, by redistributing the hormone-free forms to cortical and epidermal cells, stimulates cell elongation [17]. Likewise, hydrotropism is also regulated by ABA [18,19] but does not require the transport and redistribution of auxins [20]. The reduction of the soil water potential is detected in the root elongation zone and not in the root apex, as is the case with other tropisms [18]. This variation affects ABA-mediated signalling, specifically in the cortical cells of the elongation zone, triggering cell expansion that determines the change in the direction of root growth towards an edaphic zone of higher available water content [19]. Signalling pathways that stimulate ABA biosynthesis in response to osmotic stress involve redox signalling, Ca^2+^ signalling, and protein phosphorylation/dephosphorylation events [21]. We identified, in *I. paraguariensis* roots, various transcripts encoding Ca^2+^-dependent protein kinases (SnRK2, CML37, CDPK1, CIPK11, SYT3), negative regulators (PP2C), and others related to G-proteins.

Current and previous results confirmed that ABA is the main regulator of the *I. paraguariensis* response to water shortage [4,5]. Furthermore, we isolated, from leaves [5] and roots, two single-exon genes, *IpNCED1* (T151) and *IpNCED2* (T7), encoding the key enzyme 9-cis-epoxycarotenoid dioxygenase, which, induced by drought, enhances ABA biosynthesis. The phylogenetic analysis of the deduced protein sequences indicated that IpNCED1 and IpNCED2 are strongly related to NCED1 and NCED3 proteins from other angiosperm species [22]. Additionally, Watanabe et al. [23], working with a series of *A. thaliana* aba3 mutants, demonstrated the existence of a new ABA-independent stress-response pathway mediated by the ABA3 enzyme. Based on their results, these authors presumed that plants develop a system that, by regulating multiple pathway metabolisms with a single enzyme (ABA3), would allow them to respond globally and cope with their constantly changing environment. However, the differential accumulation of *IpNCED2* mRNA promoted by dehydration and, at the same time, the decrease in the expression of transcripts encoding ABA3 (T8) indicates that ABA mainly mediates the response to dehydration in *I. paraguariensis* plants.

ABA acts in plants by binding to the intracellular receptor protein Resistance 1/PYR1-like (PYR1/PYL), called PYLs, forming a complex with clade PP2Cs, resulting in the release of SnRK2 (sucrose non-fermenting 1-related protein kinase 2) from the inhibition exerted by PP2Cs [24,25]. Consequently, SnRK2s are, in turn, activated by other protein kinases, such as MAPKKKs, which regulate multiple physiological responses through the phosphorylation of target substrates that include ion channels, transcription factors, and transporters, among others [26]. Conversely, in the absence of ABA, PP2Cs repress SnRK2s, disrupting the signalling process. In this sense, the overexpression of ABA receptor proteins, PYR/PYL (T1), in *I. paraguariensis* roots enables the ABA-mediated signal transmission to the target proteins, including the AREB/ABF transcription factors, MYB/MYC [27], anion channels, and NADPH-dependent oxidases [28]. Many components of ABA signalling have been related to the dehydration response in woody species. For example, the overexpression of *PtPYRL1* or *PtPYRL5* increases tolerance to drought [29]; while the overexpression of the *PP2C* gene of *Populus* negatively regulates this response, increasing water loss [30,31].

Additionally, the overexpression of transcripts (T14) encoding SAUR32 (small auxin-up RNA 32), induced by auxins, reveals a connection point with ABA-mediated signalling at the root level [32]. In this context, the repression of the transcripts encoding GH3 protein, responsible for the inactivation of free auxins by conjugation with amino acids, ensures adequate hormonal homeostasis [33] to regulate root growth as a function of soil moisture. The accumulation of mRNA encoding ACC oxidase, a key enzyme in ethylene biosynthesis, would indicate that the promotion of *I. paraguariensis* root growth in response to drought is regulated by ABA, auxins, and ethylene crosstalk [34].

#### 3.2.2. Transcription Factors

The promoter region of ABA-inducible genes may contain multiple cis-elements, such as ABREs (PyACGTGG/TC), that are recognised by transcription factors (TFs), including ABA-responsive element (ABRE)-binding proteins, (AREB)/ABRE-binding factors (ABF), WRKY, NF-Ys, and MYB [35,36]. Likewise, the expression of genes involved in ABA-independent signalling pathways is controlled by AP2/ERF (APETALA2/ethylene responsive factor) and NAC (NAM, ATAF1/2, and CUC2) [37]. This study identified several TFs associated with abiotic, biotic, and combined stress responses in *I. paraguariensis* roots. MYB, bZIP, WRKY, C2H2, and NAC were the most abundant dehydration-related families.

AREBs/ABF TFs, which bind to the ABRE cis-element, belong to the bZIP family, and their expression is induced by dehydration and ABA application [36]. Water shortage strongly stimulated in *I. paraguariensis*-stressed roots the expression of an *A. thaliana* ortholog (AT5G11260, T38), encoding the ELONGATED HYPOCOTYL5 (HY5) transcription factor. HY5 acts as a master regulator that controls the expression of many genes in response to developmental, hormonal, and dynamically changing environments [38]. In addition, HY5 functions as a key transcription factor regulating root responses to high ambient temperature [39]. HY5 promotes root thermomorphogenesis by directly controlling the expression of many genes, including auxin and brassinosteroid pathway genes, to regulate the root architecture in such conditions. In addition, we identified another factor known as COP1 (constitutive photomorphogenesis 1), whose expression, promoted by HY5 [40], increased by four times in response to drought. In leaves, HY5 is also related to the induction of the *TPS1* (trehalose-6-phosphatase) gene and carbohydrate transporters of the SWEET11/12 type (sugar will be eventually transported 11/12) [41]; it is to be highlighted that both transcripts have also been stimulated in *I. paraguariensis* roots. Furthermore, HY5 regulates the expression of several IAA/auxin proteins involved in proteolysis mediated by the ubiquitin-proteasome system, necessary for lateral root initiation [42]. In this sense, we observed the induction of IAA6 and IAA29 transcripts and several mRNAs related to ubiquitination-mediated proteolysis, including UBQ3, UBQ4, UBP6, UBP8, ARI8, E3, and PAF2. Thus, these results suggest that HY5 would be involved in trehalose biosynthesis, sugar transporters, and the modulation of protein degradation that occurs in the roots in response to drought.

The WRKY family of transcription factors contains a conserved domain, which recognises the W-box (TAGACC/T), located in the promoter region of numerous biotic and abiotic stress response genes [43]. We annotated four sequences from roots, WRKY3, WRKY4, WRKY23, and WRKY33, whose expressions increased significantly by dehydration. WRKY3 and WRKY4 were induced in response to pathogens through salicylic acid-mediated signalling [44]. WRKY23 has been identified as a regulator of auxin signalling downstream of IAA14, ARF7, and ARF19 [45]. Finally, WRKY33 was related to the *A. thaliana* drought tolerance response [46] by regulating the expression of some genes involved in ethylene biosynthesis [47]. Likewise, WRKY33 can interact with bZIP proteins and other WRKY elements, promoting tissue development and connecting with the biotic stress response pathway, especially with that linked to necrotrophic fungi [48]. In addition, many members of WRKY were identified in other woody species, including *Populus*, *Pinus*, and *Vitis* [49,50,51].

In some drought-sensitive ABA-dependent genes, the ABRE cis-element is substituted, specifically by MYBRS (C/TAACNA/G) and MYCRS (CANNTG) [36]. MYB TFs are involved in response to drought, fulfilling several regulatory functions linked to wax synthesis, CBF genes, expansins, endoglucanases, ABA synthesis, and reactive oxygen species scavenging [52]. For example, in *I. paraguariensis*-stressed roots, we observed the overexpression of MYB20, MYB59, MYB73, MYB85, MIB121, and MIB305. In this context, MYB20 having ABI1 and PP2CA as target proteins would be involved in the ABA-dependent signalling [52]. MYB59 can also bind to the DRE cis-acting element, regulating cell cycle and root growth [53,54]. MYB59 also regulates the *NPF7.3* expression in *A. thaliana* in response to the availability of K^+^/NO_3_^-^, coordinating its transport to the aerial part [55]. Meanwhile, MYB73 promotes the ionic stress response [48]. Additionally, Taylor-Teeples et al. [56] reported that the secondary cell wall formation in the *A. thaliana* xylem roots would be associated with MYB20, MYB73, and MYB85 together with the AP2-EREBP, bHLH, C2H2, C2C2-GATA, and GRAS TF families. It is worth highlighting that MYB121, a central regulator of ABA-mediated signalling [57], increased by 1176 times the expression in *I. paraguariensis* roots under stressed conditions. Likewise, ATMYB71, an ortholog of MYB305, would also be associated with the greater tolerance of sesame roots to dehydration [58]. About 190 MYB TFs were identified in *Populus* [59], and several assigned functions were related to drought tolerance.

We highlight the DREB2 and NAC families of transcription factors among the ABA-independent signalling. DREB2A, DREB2B, and DREB2C belong to the petala2/ethylene-responsive factors (AP2/ERF) family, which, in turn, can act as regulators of other TFs, including the heat shock factors (HSFs) [60]. We observed a significant accumulation of HsfA2, HsfC1, and HsfB2B transcripts in *I. paraguariensis* roots, which may be related to the dehydration resilience response by regulating some heat shock proteins, with chaperone functions protecting the protein structure and folding [61]. In the same way, these TFs participate in the biosynthesis regulation of compatible osmolytes. Indeed, it has been shown that HsfA2 induces the expression of galactinol synthase and raffinose synthase in *Zea mays* [62]. 

AP2/ERF also can act as an interconnection point in the hormone signalling network. The ABA and ethylene signalling pathways are activated by a stressor, interconnecting with AP2/ERF through the ABI and EIN proteins [63]. We identified, in *I. paraguariensis* roots, the transcriptional repression of the CTR1 protein kinase, whose activity is restricted by ethylene, allowing the activation of downstream proteins that leads to the expression of ethylene response TFs [64]. Consequently, we observed an increase in ERF110 and ERF115 transcripts belonging to the ERF subfamily B4. In connection with brassinosteroids, the latter is necessary to maintain the quiescent centre in the root meristem [65].

Many members of the NAC family, induced by drought or ABA, bind to the NACRS(CGTG/A) sequence in the promoter region of dehydration-responsive genes [66]. NACs regulate drought response through ABA-dependent and independent pathways. In this sense, we observed a substantial increase in the expression of a transcript encoding NAC72/RD26 (responsive to desiccation 26), considered key in the ABA and brassinosteroids crosstalk [67,68]. Likewise, in response to the strain, an increase in mRNA, encoding the factors ATAF1, NAC14, and NAC28, was observed. ATAF1 is involved in the positive regulation of ABA synthesis through its binding to the promoter of the *NCED* gene [69]; NAC14 is linked to DNA repair in rice plants subject to drought [70]. Numerous TFs belonging to the NAC family have been isolated and characterised as woody species, including *Populus* spp. [71] and *Vitis vinifera* [72].

Finally, we identified transcripts from the HD-Zip and C2H2 families whose expression is stimulated by environmental constraints, ABA, and ethylene [73,74]. Both proteins promote root growth and prevent oxidative stress under such circumstances [75].

#### 3.2.3. TOR Signalling

Target of rapamycin (TOR) kinase has been recognised as a key developmental regulator in plants and animals [76]. The TOR signalling pathway is vital to integrate information about the cell and tissue nutrient status and energy to direct the appropriate developmental and physiological response [77]. In this way, leaf cells regulate plasmodesmata trafficking in response to changing carbohydrate availability monitored by the TOR pathway [78]. Although controversy exists regarding its function under stress conditions, reciprocal regulation was confirmed between ABA and TOR [79]. In unstressed situations, TORC1 phosphorylates the ABA receptor PYL, preventing ABA signalling. On the other hand, under stress conditions, ABA represses TOR via SnRK2 and SnRK1 kinase activities. In this circumstance, SnRK2 phosphorylates the RAPTOR protein, which causes its dissociation from TORC1 [79,80].

In response to soil water shortage, we detected an 832-fold increase in the root expression of a transcript (T32) encoding serine/threonine, a target protein for rapamycin. This fact may be related to the accumulation of glucose, translocated from the leaves as sucrose, linking root development with the source/sink relation [81]. Root glucose variation was related to the accumulation of transcripts involved in sucrose hydrolysis and the raise of its products, glucose and fructose content.

### 3.3. Metabolic Responses to Dehydration

#### 3.3.1. Non-Structural Carbohydrates

Non-structural carbohydrates (NSC) constitute an essential component of the carbon budget with which the plant must alleviate the adverse conditions generated by stress. In other words, the amount of NSC available at any given time could reflect the tree’s resilience to a drought episode. These include starch and soluble sugars consumed to produce root hairs, respiration to generate available energy, and osmotic adjustment [6].

Previously, by using the same *I. paraguariensis* genotype, we determined that, in response to water deficit, the starch and sucrose content decreases in leaves due to the restriction of CO_2_ fixation and an increase in gene expression encoding enzymes that catalyse the hydrolysis of carbohydrates [5]. In this study, we observed a reduction in the number of starch grains in the root cells and a decrease in the total polysaccharide content (Figure 4), conforming to the transcriptional stimulation of the enzymes involved in their hydrolysis. At the same time, the sucrose, glucose and fructose contents increased progressively due to the water shortage, whose magnitudes depended on the strain intensity. Thus, considering the whole plant response, we can assume that *I. paraguariensis* reallocates its photosynthates (mainly sucrose) towards roots to support their hydrotropic growth.

The up-regulation of genes encoding transcription factors and enzymes involved in its biosynthesis increased the trehalose content in the stressed roots. This fact could be interpreted as a resilience response since the trehalose accumulation could decrease the water potential of the root cells, allowing the entry of water. Additionally, trehalose could improve cells’ redox balance, helping preserve membrane functionality under severe dehydration conditions [82]. Likewise, trehalose-6-P synthase may have a regulatory role in glycolysis by the conversion of glucose-6-P into trehalose, restricting the flow of glucose [83]. Furthermore, a recent review by Fitchner and Lunn [84] highlighted the dual role that trehalose-6-P would have as a signalling molecule and homeostatic regulator of sucrose levels in plants. In this sense, trehalose-6-P regulates the production of sucrose in leaves (source), balancing the disaccharide supply in the growing organs (sink). Finally, considering that *I. paraguariensis* interacts with endogenous mycorrhizae [85], the increase in trehalose content could also serve as a carbon source for the symbiont, improving the absorption of nutrients and water [83].

#### 3.3.2. Organic Acids

The versatile nature of organic acids allows them to contribute in multiple ways to the biochemical processes that trigger a physiological response to a particular stressor [86]. For example, stress stimulates its biosynthesis and distribution in the plant; thus, multiple genes encoding enzymes involved in organic acid pathways are differentially expressed to adjust the acclimation process.

Among the organic acid plants used to alleviate water stress, Krebs Cycle (KC) intermediates and other short-chain carboxylic acids are highlighted [87]. In addition, KC intermediates and citric and malic acids, are considered good candidates to act as signalling molecules, at least in *A. thaliana*, since they reflect the redox and metabolic state of the cell and can be transported between its compartments [88]. In this context, the citric and succinic acid contents decreased significantly in stressed leaves [5], while no differences were observed in root tissues. This fact, added to the evidence at the transcriptional level, could indicate that the severity of the stress (Ψ_soil_ = −2 MPa) still does not endanger the production of reduction equivalents necessary to maintain the metabolic processes in the root environment. 

Likewise, the malic acid content increased significantly in leaves and roots, correlating with the overexpression of malate synthase, an enzyme that uses acetyl-CoA and 2-oxoglutarate as substrates; therefore, the content of the latter decreased by more than half in the stressed roots. Correspondingly, the expression of transcripts encoding malate dehydrogenase, which catalyses the conversion of malic acid into oxaloacetic acid, a precursor of the amino acid aspartate and asparagine, increased by two times.

#### 3.3.3. Amino Acids

The nitrate and protein content in correspondence with the transcriptomic profile of genes involved in the uptake of inorganic nitrogen did not show variations in *I. paraguariensis* roots subjected to water deficit. However, the activation of numerous genes encoding transcripts involved in protein synthesis and degradation was observed. These results suggest an apparent balance of protein metabolism, optimising the nitrogen available to the tissue.

The total amino acid content rise was mainly attributed to increased branched-chain amino acids. In addition, a positive transcriptional variation was observed in the respective biosynthesis pathways where they doubled (Leu and Val) or quadrupled (Ile) their endogenous levels in response to drought. Similarly, the alanine content, which can originate pyruvate by transamination with glyoxylate or 2-oxoglutarate, increased significantly in roots. The increase in the branched and related amino acid contents, such as Ala and Thr, has been associated with dehydration tolerance in numerous plant species, contributing to osmotic adjustment [89] and as substrates for alternative respiration pathways [90]. Likewise, these compounds could be linked to the signalling process mediated by the TOR proteins [91]. Glutamic acid content was reduced by half in stressed roots, while its derivative ornithine, the precursor or the polyamines putrescine and spermidine, accumulated more than thirteen times. This fact could be related to the transcriptional control of putrescine on NCED [92]. Finally, as part of an integrated response of the plant, the eventual excretion of these biomolecules through root exudate should be considered an important acclimation mechanism [88].

## 4. Materials and Methods

### 4.1. Plant Material, Drought Assay, and Sample Collection

Two-year-old *Ilex paraguariensis* St. Hil. cv SI-49 plants from rooted cuttings were grown in 3 L tube-like containers filled with lateritic red soils (Alfisols) under greenhouse conditions. One week before starting the experiment, plants were pruned and transferred to a room with controlled environmental parameters, 27 ± 1/22 ± 2 °C day/night temperatures, 50–55% relative humidity, and a 14 h photoperiod (420 μmol photons·m^−2^·s^−1^, provided by mercury lamps). After acclimation, pots were irrigated until the soil water potential (Ψ_soil_) reached field capacity (CC, Ψ_soil_ ≈ −0.04 MPa) and were subsequently subjected to a continuous soil drying episode by withholding water from pots until Ψ_soil_ at pre-dawn reached −2 MPa (severe stress). A rewatering treatment (at Ψ_soil_= −2 Mpa) was also included. For the control treatment, the soil water content was kept at field capacity by restoring water loss by transpiration daily. Plants from identical genotypes and ages were used in all treatments. Pots were covered with aluminium foil to prevent water loss by evaporation from the soil surface. This experiment was carried out under similar environmental conditions for 22 days. The soil water potential was determined using a psychrometer HR-33T with a PST-55 thermocouple (Wescor Inc., Logan, UT, USA). For root osmotic potential measurement, the roots were re-hydrated to constant fresh weight by placing them in a beaker of distilled water under controlled environmental conditions, then placed in a syringe, frozen in liquid nitrogen, and kept at −80 °C pending further analysis. Syringes were thawed until samples reached room temperature, and the osmotic potential of expressed sap was measured with a C-52 thermocouple [93]. Root relative water content [RWC % = (fresh weight − dry weight)/(turgid weight − dry weight) × 100] was measured at midday. Root growth was quantified indirectly by using the electric capacitance method [94]. For the scanning electron microscopy observation, the fixed samples were dehydrated using an acetone series, dried with CO_2_ using the critical point technique, and coated with gold and palladium. A JEOL scanning electron microscope (JLV 5800) operated at 20 kv (Universidad Nacional del Nordeste, Argentina) was used to examine and photograph the root tips.

Three biological replicates were used per treatment. For transcriptomic and metabolomic analyses, primary roots were harvested at midday, immediately frozen in liquid nitrogen, and kept at −80 °C until processing.

### 4.2. RNA Isolation, cDNA Synthesis, and RNA-Seq Library Preparation

Total RNA samples were obtained using the Spectrum™ Plant Total RNA Kit (Sigma-Aldrich Inc., St. Louis, MO, USA) with DNase I treatment, following the manufacturer’s instructions [95]. RNA integrity was performed using the Agilent 2100 Bioanalyzer system with an RNA nano/pico chip platform (Agilent Technologies, Santa Clara, CA, USA). Only samples with an RIN value ≥ 7 (RNA Integrity Number) were used in the following procedures. Purity was assessed by the A260/A280 and A260/A230 absorption ratios with a NanoDrop™ 2000 spectrophotometer (Thermo Fisher Scientific, Wilmington, DE, USA), and concentrations were determined at 260 nm.

For the qPCR reactions, cDNAs were synthesised from the RNA samples using the ImProm-II™ reverse transcription system (Promega Corp., Madison, WI, USA), following the manufacturer’s instructions, with oligo(dT)20 as primers. Likewise, six cDNA libraries from both treatments were prepared as described in the TruSeq^®^ RNA Sample Preparation Kit (Illumina Inc., San Diego, CA, USA) and sequenced on an Illumina HiSeq 1500 platform to produce paired-end reads (2 × 100 bp).

### 4.3. RNA-Seq Data Processing, de Novo Assembly, and Gene Expression Analysis

Raw-read libraries produced by the RNA-seq experiment were assessed for quality using the FastQC tool (http://www.bioinformatics.babraham.ac.uk/projects/fastqc/). Low-quality reads were discarded, and adapters were clipped using Trimmomatic v 0.36 [96]. Then, employing the FLASH software [97], the remaining high-quality reads were merged into a longer sequence only if both paired reads overlapped each other at their ends by at least 20 bases. The final set of reads (paired-ends and longer singles after merging) were assembled into a de novo transcriptome by the Trinity package, version 2.0.6 [98], with parameters—min_kmer_cov 3 and min_glue 5. Using the BLASTn tool, sixty-two sequences previously obtained by the Sanger method (available in GenBank) were searched among the sequences comprising the assembled transcriptome to validate the assembled transcript sequences (Supplementary Table S1).

High-quality filtered reads were mapped onto the de novo assembled transcriptome using Bowtie 2 [99] and HTSeq-count [100] software to count the aligned reads overlapping each transcript. The differential expression analysis between drought-stressed and control plants was performed using the normalisation method by TMM (trimmed mean of M values) and negative binomial distribution, as implemented in the Bioconductor edgeR package [101]. The sequences that present an expression change, |FC| ≥ 4 and FDR < 0.05, were considered differentially expressed transcripts (DETs) in the roots of stressed plants. This criterion was changed to |FC| ≥ 2 and FDR < 0.001 for specific metabolic pathways of interest to define a sequence as DET.

### 4.4. Functional Annotation of Transcripts and Metabolic Pathways Analysis

All assembled transcripts were annotated by sequence similarity comparisons using BLASTx against NCBI non-redundant protein sequences (nr) and the base UniProtKB/Swiss-Prot databases, with an e-value cutoff of 10^−5^ using the Blast2Go PRO 5 software [102]. GO (Gene ontology) terms were also assigned using this software. In addition, Trinotate v2.0.2 suite (https://trinotate.github.io/, accessed on 3 June 2018) was used to identify protein domains (Pfam database), to predict sequences of signal peptides (SignalP) and transmembrane domains (TMHMM), and to obtain annotations from the EggNOG database.

Additionally, the functional enrichment of metabolic pathways was performed through diverse software and following different strategies. Using the Blast2Go PRO 5 software, the differentially expressed transcripts (DETs) were annotated as mentioned above, and subsequently, a Fisher’s exact test was performed for both the induced and the repressed sequences obtained in the differential expression analysis separately (settings: filter value: 0.05; filter mode: FDR; one-tailed). The graphs corresponding to the different GO categories were made using the Go Graph tool. Using another approach, the MapMan software [103] allowed the representation of the biological processes through coloured diagrams, where the colour intensity is proportional to the transcript expression change. In this context, each DET was assigned a Bin-code using the Mercator software [104]. Then, each DET annotated with its respective log_2_FC was incorporated into the MapMan program, and the different schemes of biological processes were generated. In the case of the KEGG Pathway (Kyoto Encyclopedia of Genes and Genomes) [105], the functional annotation (KO number assignment) of each DET was performed using the KASS (KEGG Automatic Annotation Server) tool, with all available organisms of the plant kingdom as the gene data set. Subsequently, the involved metabolic pathways were obtained using the set of KO numbers obtained and the KEGG Mapper-Search pathway tool (http://www.genome.jp/kegg/tool/map_pathway1.html).

Finally, to generate protein–protein interaction networks enriched in transcription factors, the open reading frame (ORFs) from induced transcripts were used as input in the STRING Search Version 10.5 bioinformatics tool (https://string-db.org/cgi), *A. thaliana* was used as a reference. In addition, to increase the sensitivity and specificity in the annotation of TFs, the induced DET sequences were also compared by BLASTx with all the protein sequences of TFs included in the published PlantTFDB 4.0 database [106].

All transcripts mentioned in the ‘Discussion’ section are described in Supplementary Table S5 and represented in the text as T1, T2, ……, Tn.

### 4.5. Quantitative PCR Analysis

Each reaction mixture of 15 μL was assembled with 10 ng cDNA, 7.5 μL 2x SYBR Select Master Mix (Applied Biosystems, Foster City, CA, USA), and 300 nM of the corresponding primer pair. Each reaction was run in technical triplicate using a 7500 Real-Time PCR System (Applied Biosystems, Foster City, CA, USA) with an initial denaturation at 95 °C for 10 min, 40 cycles of 95 °C for 15 s, and 60 °C for 1 min. The specificity of the amplicons was assessed by a melting curve analysis after the qPCR run (by heating from 60 to 95 °C). For each primer, the real-time PCR amplification efficiency was determined by five-fold serial dilutions of the cDNAs [107] in a range of 25, 5, 1, 0.2, and 0.04 ng. Each dilution was performed in triplicate. The RNA polymerase-associated protein rtf1 (RTF) gene (GenBank: KU886201) was employed as an internal control [108], and no template controls were included. The relative expression value by the delta-delta CT method was calculated from three plants (each in technical triplicate) and expressed as the fold change relative to expression in control plants, to which the value 1 was assigned.

### 4.6. Metabolite Profiling Analysis

Metabolite extraction was performed using ground lyophilised root samples, followed by the addition of the appropriate extraction buffer as described by Gibon et al. [109]. The sucrose, fructose, and glucose levels were determined following the procedures described by Fernie et al. [110] and Lisec et al. [111]. Total amino acids from the soluble fraction, total protein, and starch contents were quantified following the methodology described by Cross et al. [112]. Malate and fumarate levels were determined with the method previously described by Nuness-Nesi et al. [113]. The total soluble phenol content was quantified spectrophotometrically using the Folin–Ciocalteu method, with tannic acid as standard. Organic acids and amino acids were determined as described by Lisec et al. [111]. Metabolite extraction, derivatisation, standard addition, and sample injection in a TruTOF gas chromatography–mass spectrometry (GC–MS) system were performed according to Osorio et al. [114]. The mass spectra were cross-referenced with those in the Golm Metabolome Database [115]. The values are the mean of three plants and are expressed as the fold change respectively to the control. Significant differences were determined by *t*-test (*p* < 0.05). The metabolomic profile data set was subjected to a principal component analysis (PCA) to find a correlation between metabolic profiles and treatments. A biplot graph was generated using the XLSTAT plugin of Excell 2010 (Microsoft^®^).

## 5. Conclusions

**Figure 6:**
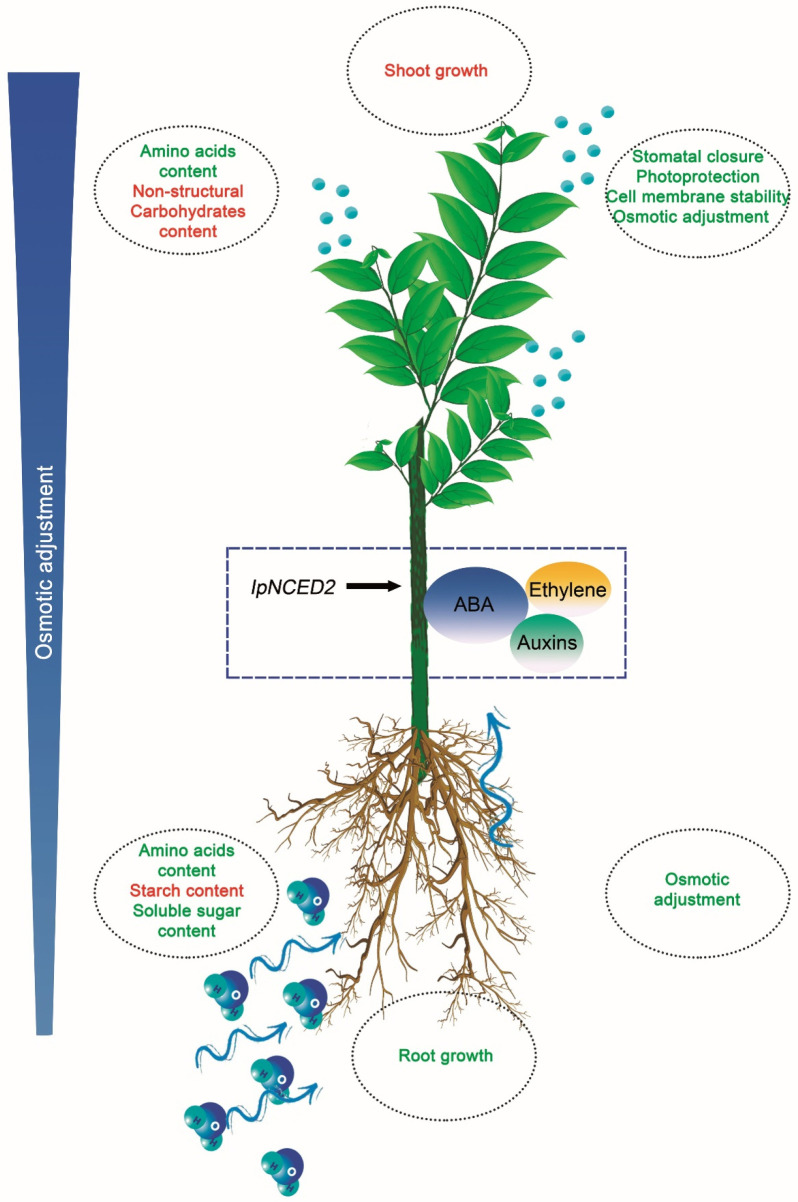
*Ilex paraguariensis* response to water shortage. Schematic diagram of the morphological, physiological, and biochemical changes in the whole plant under a severe drought episode. Parameters promoted or repressed in response to the strain are highlighted in green and red, respectively.

From the molecular, biochemical, physiological, and morphological data that arise in leaves [5] and roots, it is feasible to integrate the signalling and response processes of the whole plant to water shortage (Figure 6) as follows: (i) *Ilex paraguariensis* modifies its morphology to guarantee the uptake of resources in limited situations. Intricate mechanisms address such plasticity, including perception, signal integration, and response to the strain. In the aerial part of the plant, this is related to optimising light absorption and stress response, while root growth is required to preserve water and nutrient supplies under limiting conditions. (ii) *I. paraguariensis* responds early to the soil water deficit by displaying a typical isohydric behaviour based on the strict regulation of stomatal conductance modulated by rapid changes in the soil’s and roots’ hydraulic conductivity. As the stress intensifies, it deploys a second defense mechanism based on increased compatible osmolytes in leaf and root cells to maintain the water flow through the soil-atmosphere continuum. In addition, this resilience mechanism allows them to preserve cell membrane functionality and protect the photosynthetic apparatus. At the same time, morphological variations are observed that promote the differentiation and growth of adventitious roots, increasing the absorption capacity of water and nutrients. (iii) The expression of *IpNCED2* (GenBank MW047100), encoding 9-cis epoxy carotenoid dioxygenase (NCED), is further induced by osmotic stress. The rise in the leaf ABA content confirmed its up-regulation. Likewise, we verified that *IpNCED2* is expressed in leaves and roots. This fact, added to the isolation of several transcripts linked to the signalling process, indicates that ABA regulates the response of *I. paraguariensis* to drought, being the promotion of root growth, a process regulated by its interaction with ethylene and auxins. (iv) The transcriptomic variation produced changes mainly in non-structural sugar, organic acid, and amino acid content, improving *I. paraguariensis* resilience.

## Figures and Tables

**Table 1:** Number of enriched functions for each GO category according to Fisher’s exact test method.

GO Domains				
	Biological Process	Molecular Function	Cellular Component	Total
Enriched functions by up-regulated genes.	44	28	12	84
Enriched functions by down-regulated genes.	162	99	27	288

## Data Availability

We stored the raw sequence in the Sequence Read Archive of The National Center for Biotechnology Information (NCBI), and they are identified with the BioProject accession number PRJNA375923.

## Supplementary Materials

The following are available online at https://www.mdpi.com/article/10.3390/plants12132404/s1, Figure S1: Volcano plot of differentially expressed transcript analysis from RNA-seq data, Figure S2: Gene expression correlation analysis, Figure S3: Enriched GO terms of the Biological Process category, up-regulated transcripts by drought, Figure S4: Enriched GO terms of the Biological Process category, down-regulated transcripts by drought, Figure S5: Enriched GO terms of the Cellular Component category, up-regulated transcripts by drought, Figure S6: Enriched GO terms of the Cellular Component category, down-regulated transcripts by drought, Figure S7: Enriched GO terms of the Molecular Function category, up-regulated transcripts by drought, Figure S8: Enriched GO terms of the Molecular Function category, down-regulated transcripts by drought, Figure S9: Transcription factor families expressed in stressed roots, Figure S10: Interaction networks of up-regulated transcription factors, Figure S11: KEGG diagram of genes associated with starch and sucrose metabolism, Figure S12: KEGG diagram of genes associated with galactose metabolism, Figure S13: KEGG diagram of genes linked to glycolysis and gluconeogenesis pathways, Figure S14: KEGG diagram of genes associated with valine, leucine, and isoleucine biosynthesis, Figure S15: Root metabolic profiles principal component analysis from non-stressed (C1, C2, and C3) and stressed (S1, S2 and S3) plants, Table S1: Quality assessment determination of de novo transcriptome assemblies using BLASTn, Table S2: Transcript level quantification by real-time qPCR, Table S3: Functions enriched in up-regulated transcripts according to Fisher’s Exact Test, Table S4: Functions enriched in down-regulated transcripts according to Fisher’s Exact Test, Table S5: Functional description of transcripts cited in the Results and Discussion sections, Table S6: Primers used for transcript level quantification by real-time qPCR.
