# Supplementary material for: Integrating Transcriptional, Metabolic, and Physiological Responses to Drought Stress in Ilex paraguariensis Roots
Source: Plants (Basel). 2023 Jun 21;12(13):2404. doi: 10.3390/plants12132404 (PMC10346965; doi:10.3390/plants12132404)
Supplement: Supplementary file 1 [file plants-12-02404-s001.zip › plants-2374140-supplementary.pdf]

Integrating Transcriptional, Metabolic, and Physiological Responses to Drought Stress in *Ilex paraguariensis* Roots

Supplementary Materials

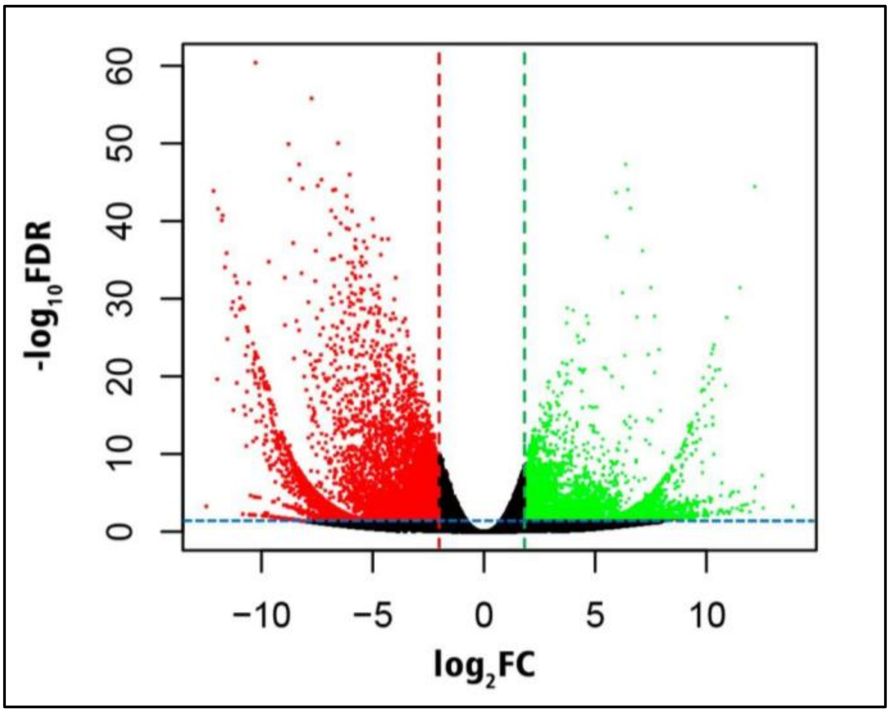

Supplementary Figure S1. Volcano plot of differentially expressed transcript analysis from RNA-seq data. The Log2 of expression Fold Change (stressed vs control plants) is represented in the X-axis, and the negative Log10 of FDR (false discovery rate) is illustrated in the Y-axis. The red and green vertical dotted lines show log2 (FC) < -2 and log2 (FC) > 2, respectively. The dashed light-blue line shows where FDR = 0,001. Up-regulated transcripts are reported as green dots, and down-regulated transcripts are red dots. Not differentially expressed transcripts are represented as black dots.

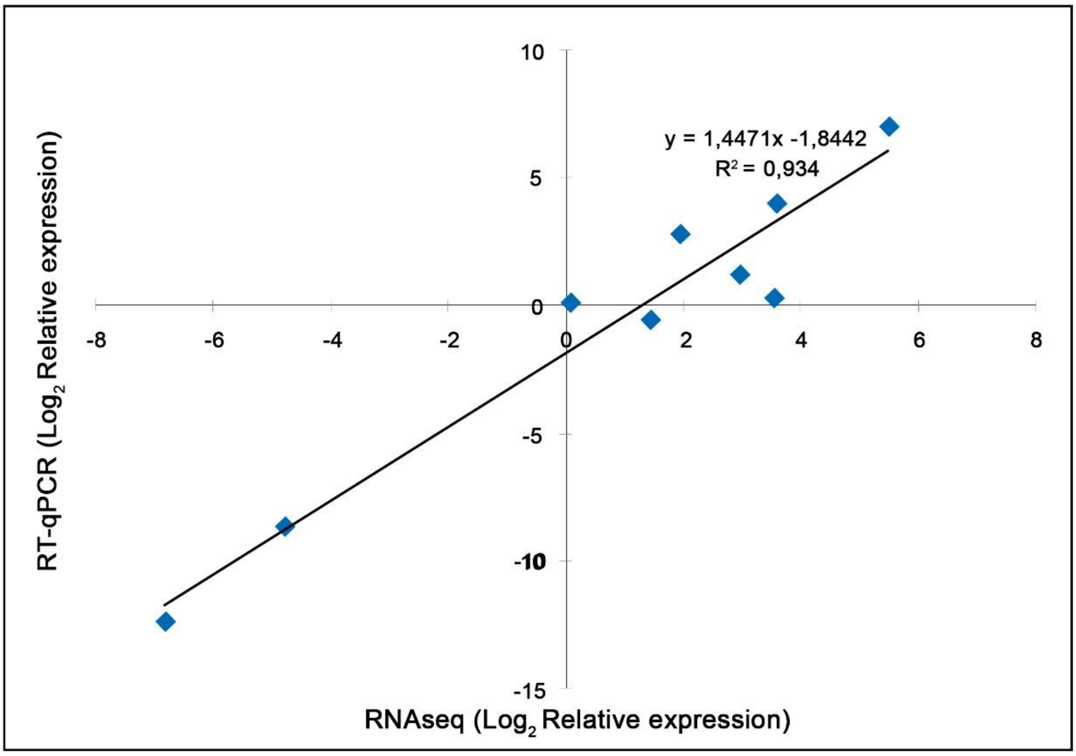

Supplementary Figure S2. Gene expression correlation analysis. The expression level correlation of nine transcripts (three biological replicates) was determined by RNAseq bioinformatics data analysis and reverse transcription followed by quantitative real-time PCR (RT-qPCR). The *RTF* gene was employed as an internal control.

Biological Process GO terms; up-regulated transcripts by drought.

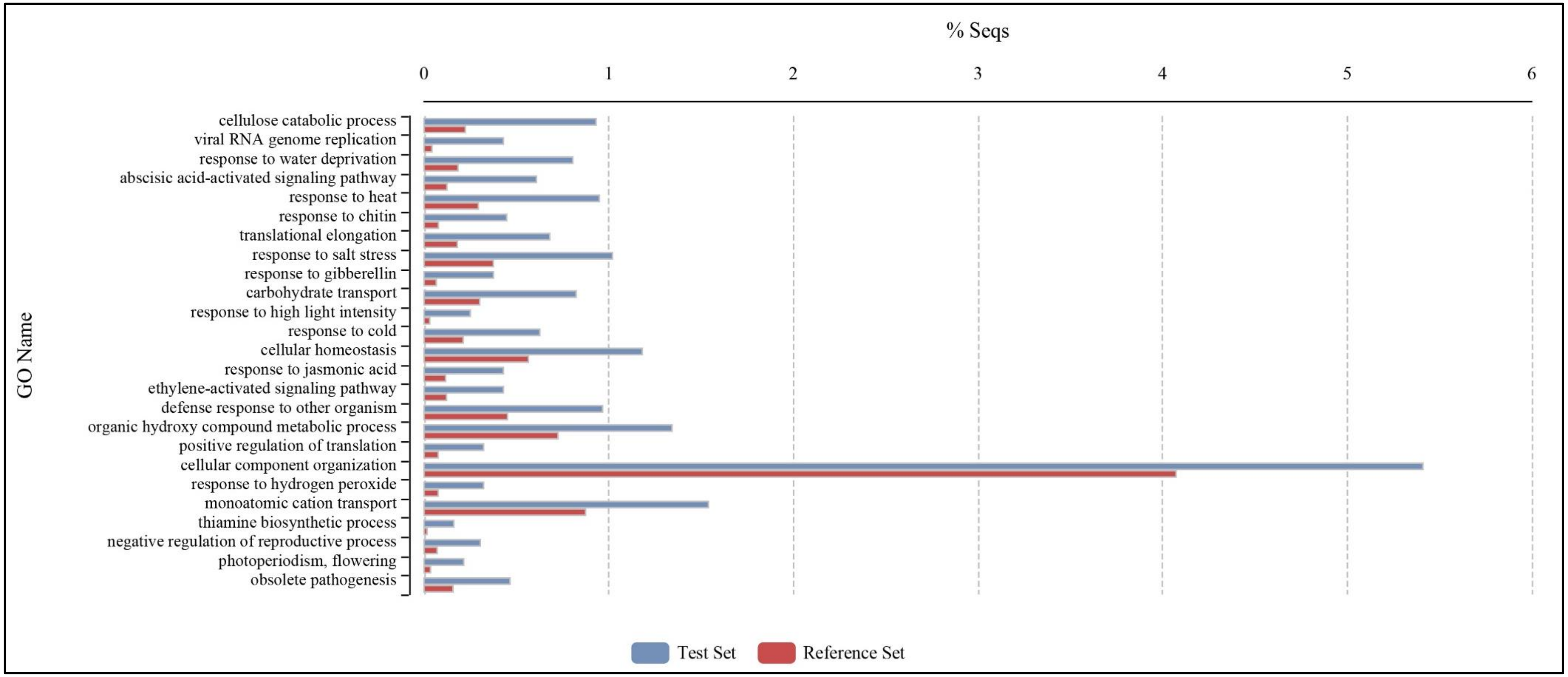

Supplementary Figure S3. Enriched GO terms of the Biological Process category, calculated by Fisher's Exact Test. The x-axis shows the sequence percentage, and the y-axis shows the significant enrichment GO terms. All the annotated transcripts represent the Reference Set (red bars), whereas the Test Set (blue bars) is represented by the up-regulated transcripts by drought (Log_2 FC $\geq$ 2, and FDR $\leq$ 0.001). Only the 25 terms with the lowest FDR are represented in the chart.

Biological Process GO terms; down-regulated transcripts by drought.

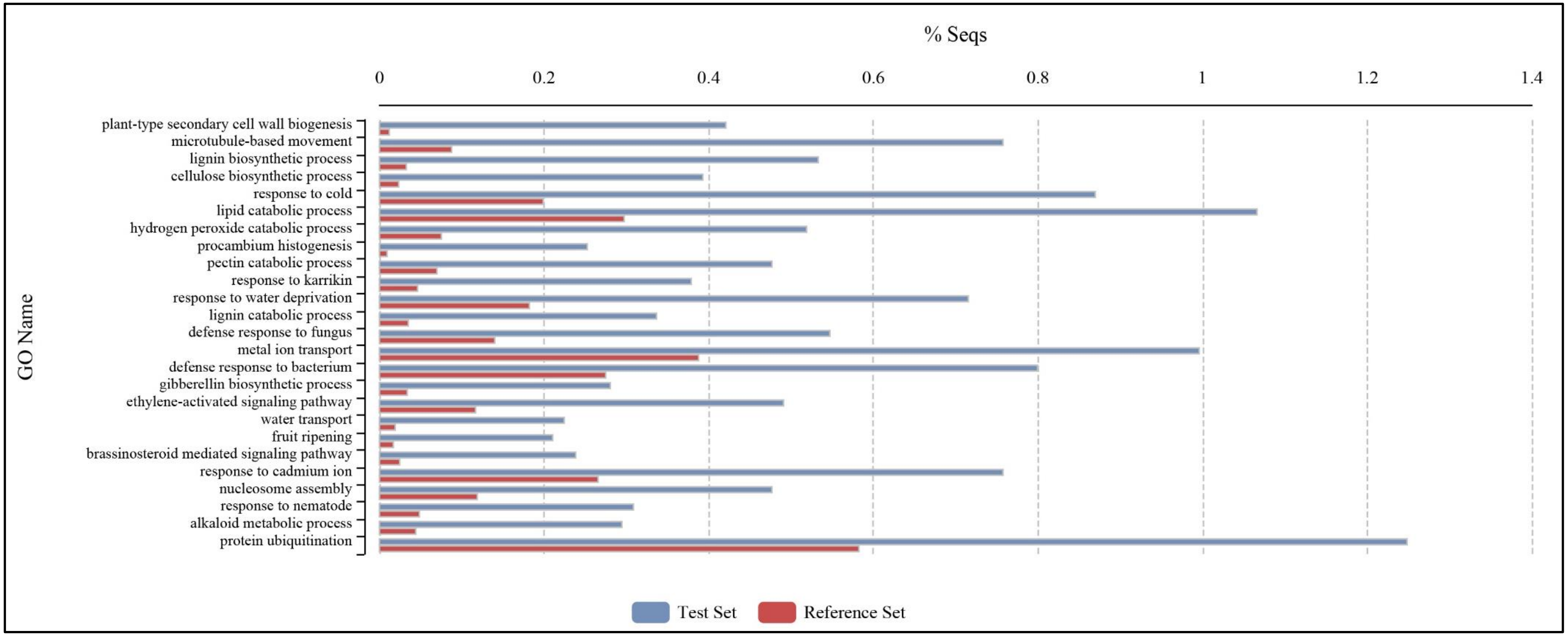

Supplementary Figure S4. Enriched GO terms of the Biological Process category, calculated by Fisher's Exact Test. The x-axis shows the sequence percentage, and the y-axis shows the significant enrichment GO terms. All the annotated transcripts represent the Reference Set (red bars), whereas the Test Set (blue bars) is represented by the down-regulated transcripts by drought (Log_2 FC $\leq$ -2, and FDR $\leq$ 0.001). Only the 25 terms with the lowest FDR are represented in the chart.

Cellular Component GO terms; up-regulated transcripts by drought.

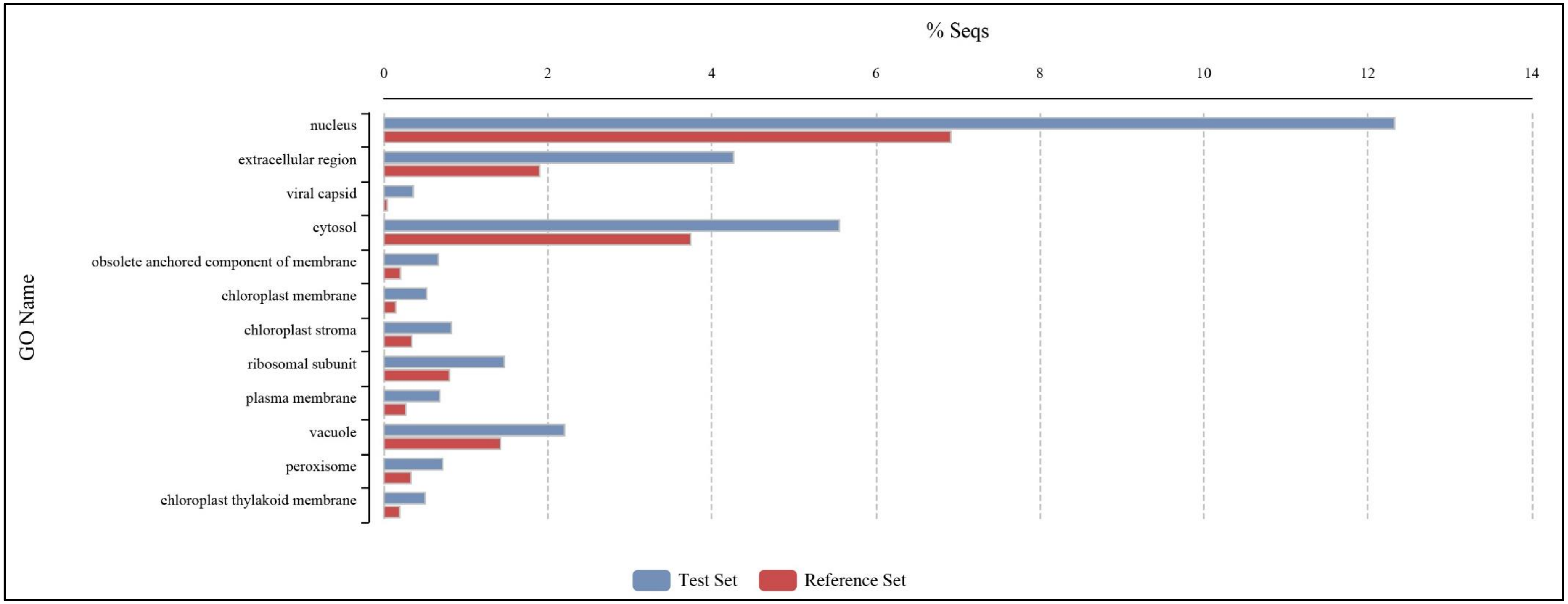

Supplementary Figure S5. Enriched GO terms of the Cellular Component category, calculated by Fisher's Exact Test. The x-axis shows the sequence percentage, and the y-axis shows the significant enrichment GO terms. All the annotated transcripts represent the Reference Set (red bars), whereas the Test Set (blue bars) is represented by the up-regulated transcripts by drought (Log_2 FC ≥ 2, and FDR ≤ 0.001).

Cellular Component GO terms; down-regulated transcripts by drought.

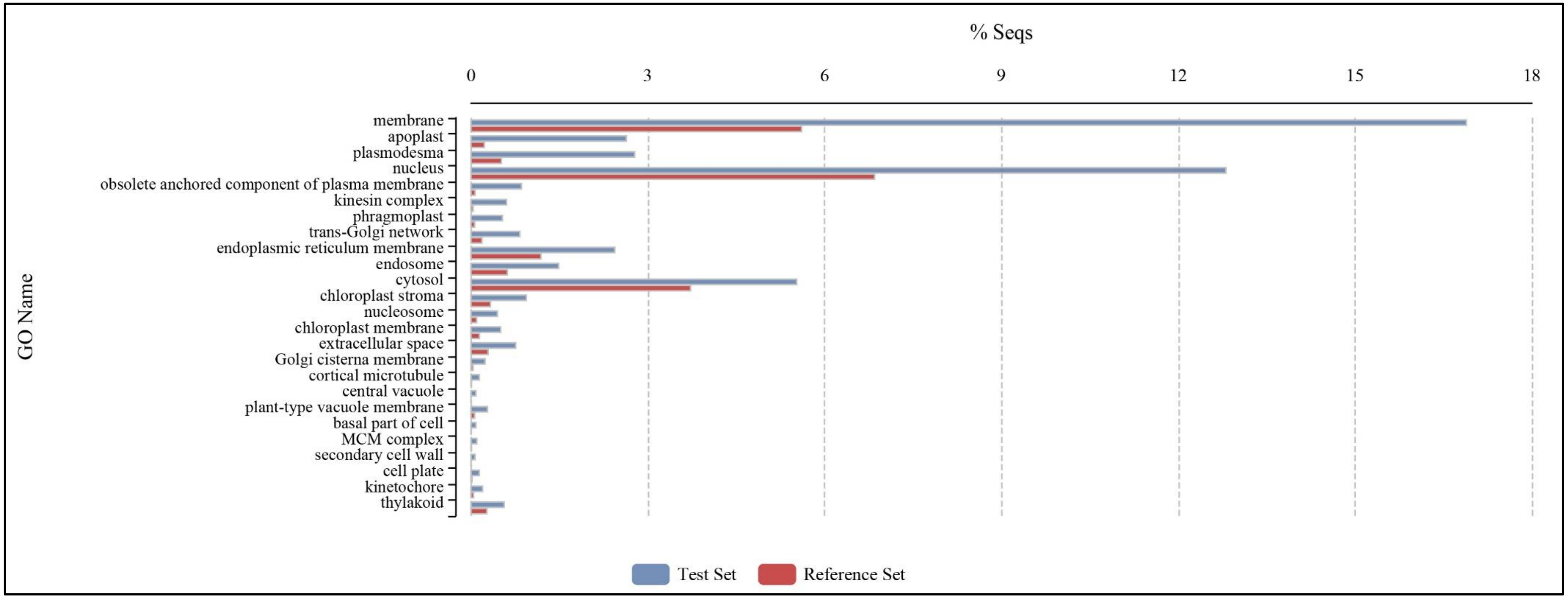

Supplementary Figure S6. Enriched GO terms of the Cellular Component category, calculated by Fisher's Exact Test. The x-axis shows the sequence percentage, and the y-axis shows the significant enrichment GO terms. All the annotated transcripts represent the Reference Set (red bars), whereas the Test Set (blue bars) is represented by the down-regulated transcripts by drought (Log_2 FC $\leq$ -2, and FDR $\leq$ 0.001). Only the 25 terms with the lowest FDR are represented in the chart.

Molecular Function GO terms; up-regulated transcripts by drought.

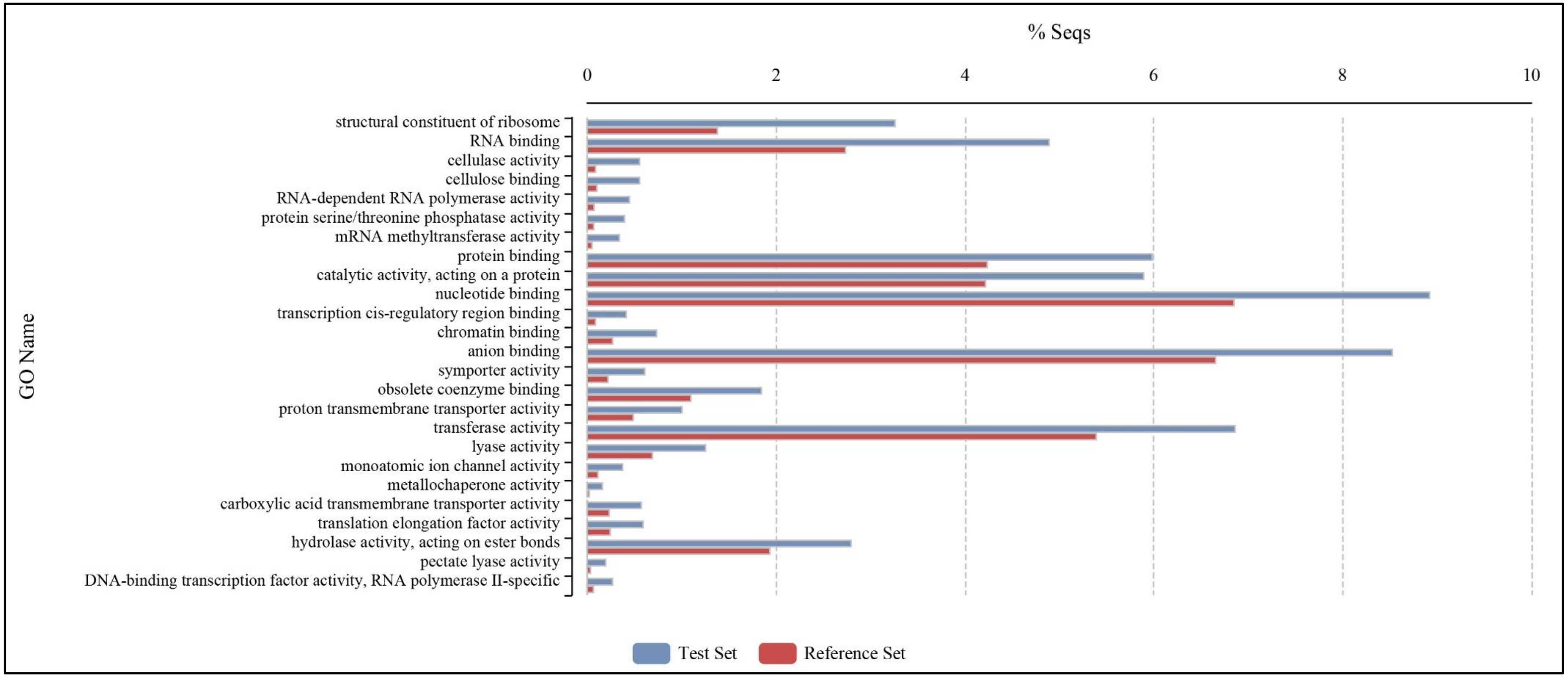

Supplementary Figure S7. Enriched GO terms of the Molecular Function category, calculated by Fisher's Exact Test. The x-axis shows the sequence percentage, and the y-axis shows the significant enrichment GO terms. All the annotated transcripts represent the Reference Set (red bars), whereas the Test Set (blue bars) is represented by the up-regulated transcripts by drought (Log_2 FC ≥ 2, and FDR ≤ 0.001). Only the 25 terms with the lowest FDR are represented in the chart.

Molecular Function GO terms; down-regulated transcripts by drought.

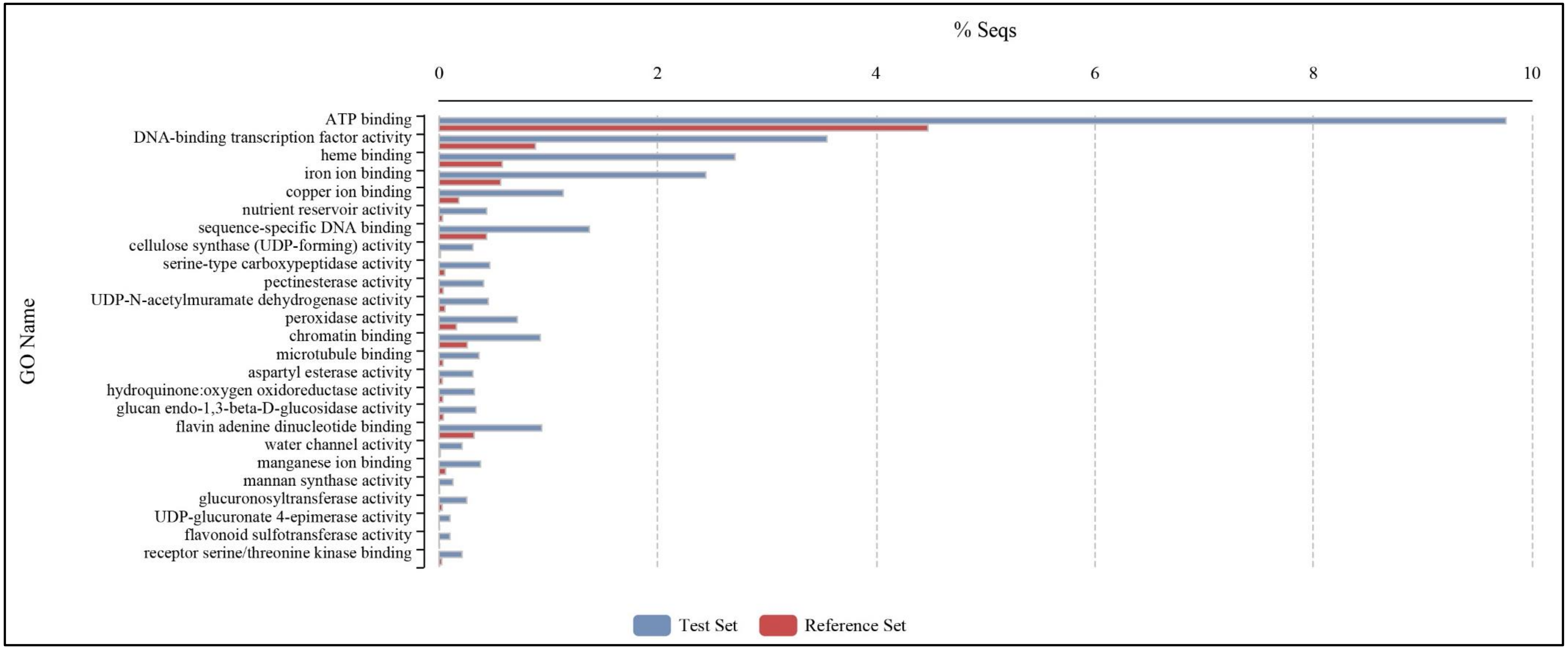

Supplementary Figure S8. Enriched GO terms of the Molecular Function category, calculated by Fisher's Exact Test. The x-axis shows the sequence percentage, and the y-axis shows the significant enrichment GO terms. All the annotated transcripts represent the Reference Set (red bars), whereas the Test Set (blue bars) is represented by the down-regulated transcripts by drought (Log_2 FC ≤ -2, and FDR ≤ 0.001). Only the 25 terms with the lowest FDR are represented in the chart.

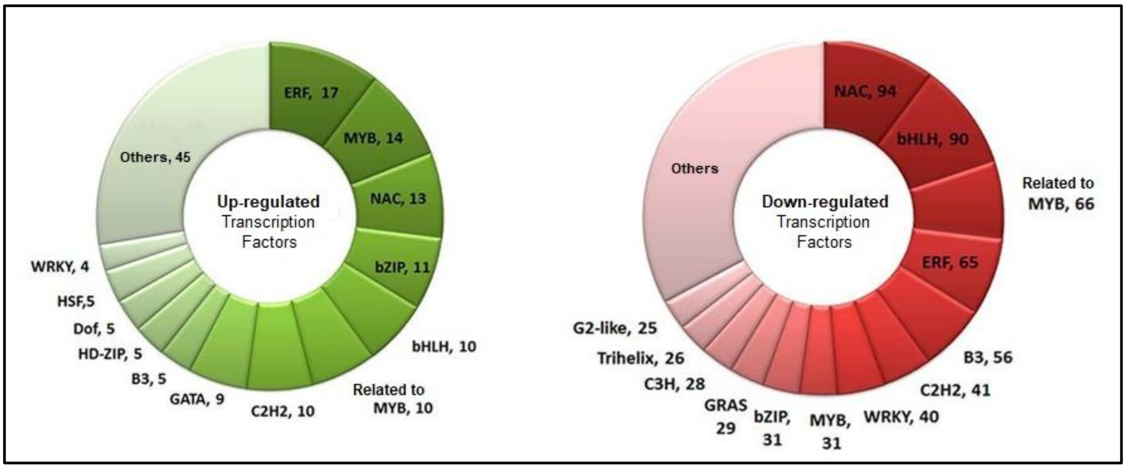

Supplementary Figure S9. Transcription factor (TFs) families expressed in stressed roots. TFs were annotated by homology with the PlantTFDB v4.0 database.

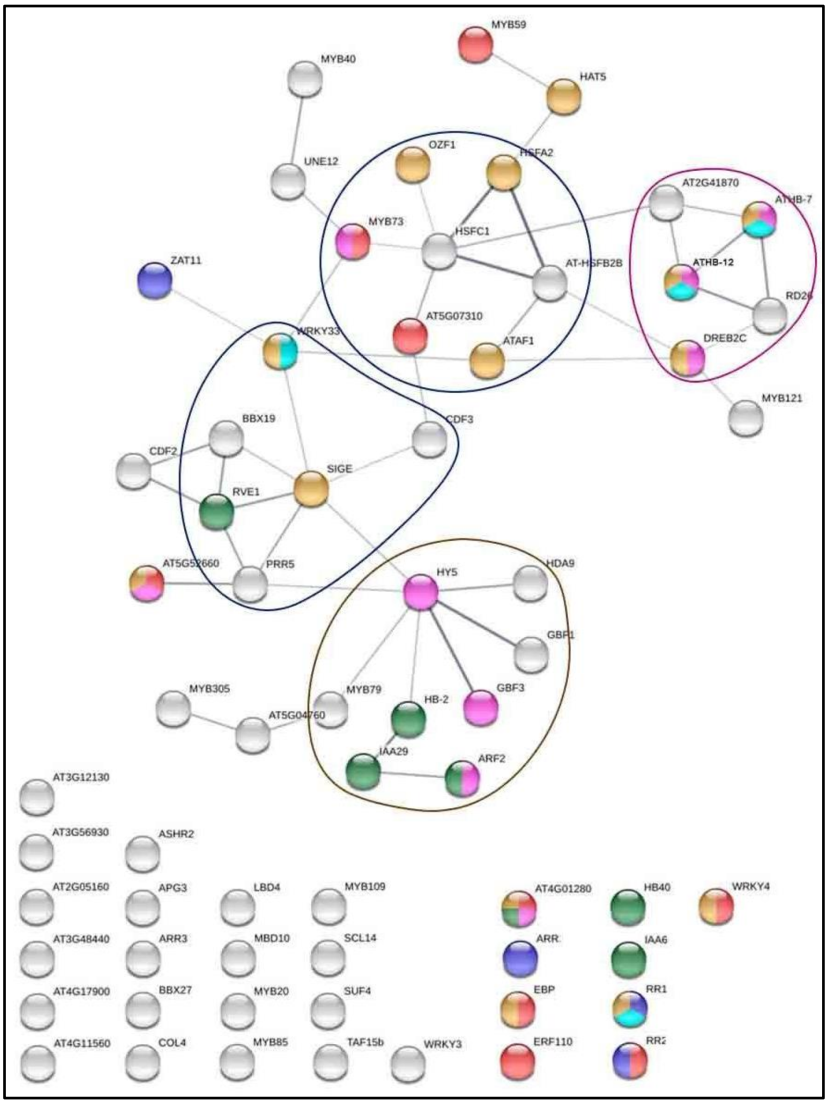

Supplementary Figure S10. Interaction networks of up-regulated transcription factors using the STRING 10.0 bioinformatics tool. The analysis of functional associations was carried out with the *Arabidopsis thaliana* database. The width line indicates the type and reliability of interaction evidence for a confidence value of 0.4. The circles are coloured according to their participation in biological processes: responses to ethylene (red), ABA (pink), auxins (green), water deprivation (light blue), stress (light brown) and root development (blue).

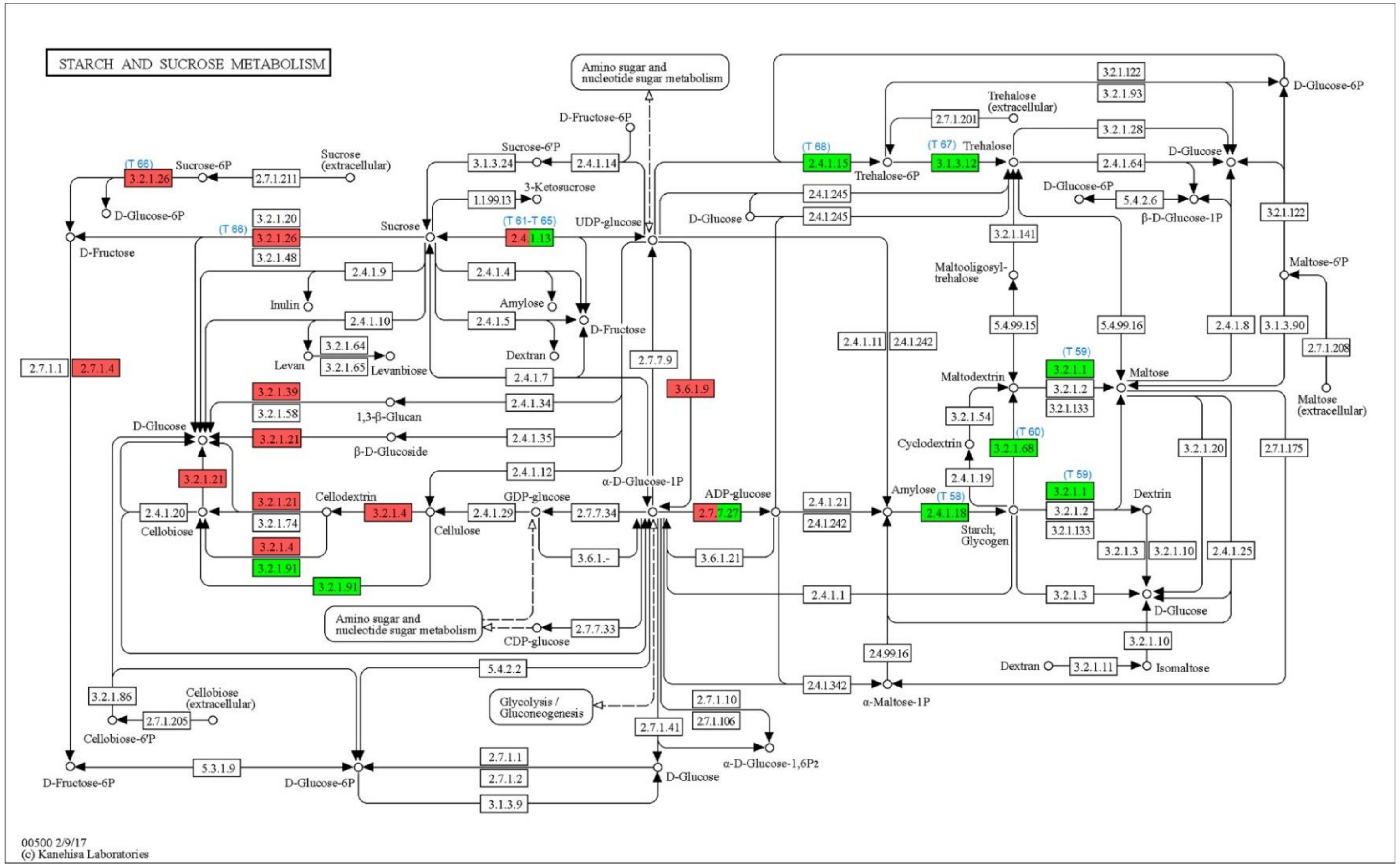

Supplementary Figure S11. KEGG diagram of genes associated with starch and sucrose metabolism. The boxes represent enzymes and contain the respective EC numbers (Enzyme Commission number). The transcript which encodes the enzyme is indicated between blue brackets. Green and red indicate increased and decreased expression (FC ≥ 2 or FC ≤ -2, FDR < 0.001). EC 2.4.1.13: sucrose synthase (T61-T65). EC 2.4.1.15: trehalose 6-phosphate synthase (T68). EC 2.4.1.18: 1,4-alpha-glucan branching enzyme (T58). EC 2.7.1.4: fructokinase. EC 2.7.7.27: glucose-1-phosphate adenylyl transferase. EC 3.1.3.12: trehalose 6-phosphate phosphatase (T67). EC 3.2.1.1: alpha-amylase (T59). EC 3.2.1.4: endoglucanase. EC 3.2.1.21: beta-glucosidase. EC 3.2.1.26: beta-fructofuranosidase (T66). EC 3.2.1.39: glucan endo-1,3-beta-D-glucosidase. EC 3.2.1.68: isoamylase (T60). EC 3.2.1.91: cellulose 1,4-beta-cellobiosidase. EC 3.6.1.9: phosphodiesterase family member.

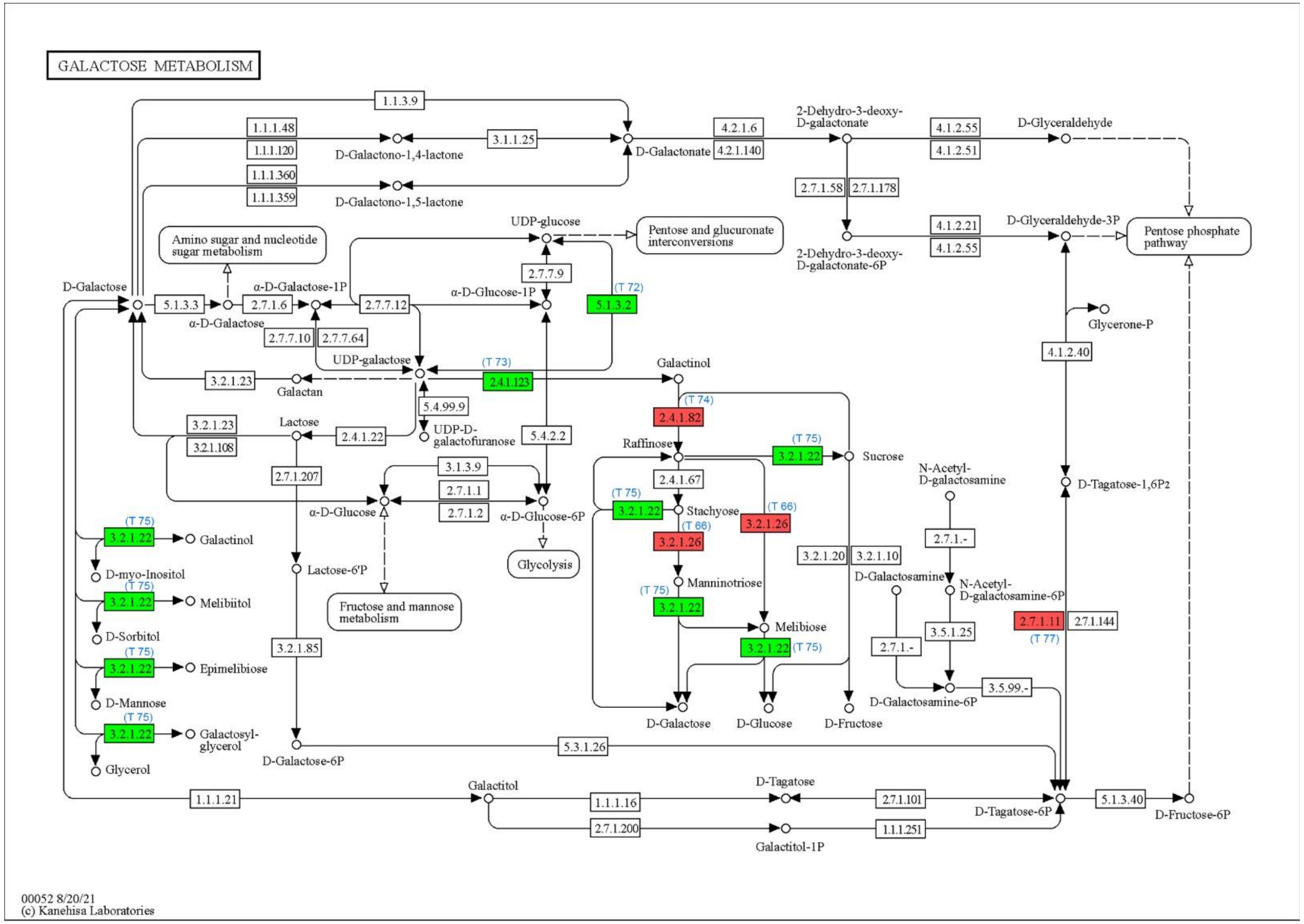

Supplementary Figure S12. KEGG diagram of genes associated with galactose metabolism. The boxes represent enzymes and contain the respective EC numbers (Enzyme Commission number). The transcript which encodes the enzyme is indicated between blue brackets. Green and red indicate increased and decreased expression (FC ≥ 2 or FC ≤ -2, FDR < 0.001). EC 2.4.1.82: raffinose synthase (T 74). EC 2.4.1.123: inositol 3-alpha-galactosyltransferase (T 73). EC 2.7.1.11: 6-phosphofructokinase 1 (T77). EC 3.2.1.22: alpha-galactosidase (T 75). EC 3.2.1.26, beta-fructofuranosidase (T 66). EC 5.1.3.2: UDP-glucose 4-epimerase (T 72).

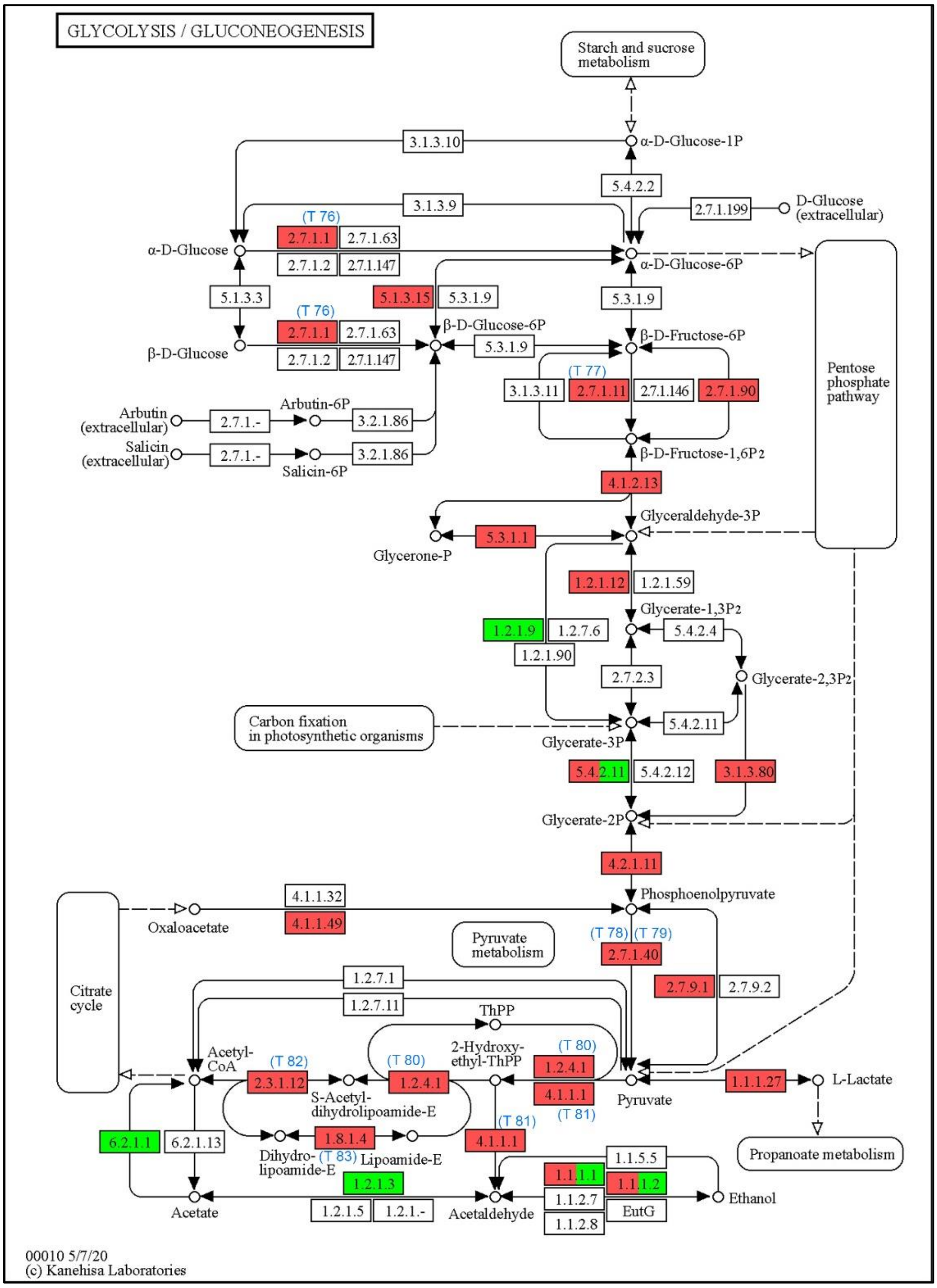

Supplementary Figure S13. KEGG diagram of genes linked to glycolysis and gluconeogenesis pathways. The boxes represent enzymes and contain the respective EC numbers (Enzyme Commission number). The transcript which encodes the enzyme is indicated between blue brackets. Green and red indicate increased and decreased expression (FC ≥ 2 or FC ≤ -2, FDR < 0.001). EC 1.1.1.1: alcohol dehydrogenase. EC 1.1.1.2: alcohol dehydrogenase ($NADP^+$). EC 1.1.1.27: L-lactate dehydrogenase. EC 1.2.1.9: glyceraldehyde-3-phosphate dehydrogenase ($NADP^+$). EC 1.2.1.3: aldehyde dehydrogenase (NAD^+). EC 1.2.1.12: glyceraldehyde 3-phosphate dehydrogenase (phosphorylating). EC 1.2.4.1: pyruvate dehydrogenase E1 component (T80). EC 1.8.1.4: dihydrolipoamide dehydrogenase (T83). EC 2.3.1.12: pyruvate dehydrogenase E2 component (dihydrolipoyllysine-residue acetyltransferase) (T82). EC 2.7.1.1: hexokinase (T76). EC 2.7.1.11: 6-phosphofructokinase (T77). EC 2.7.1.40: pyruvate kinase (T78, T79). EC 2.7.1.90: diphosphate-dependent phosphofructokinase. EC 2.7.9.1: pyruvate, orthophosphate dikinase. EC 3.1.3.80: 2,3-bisphosphoglycerate 3-phosphatase. EC 4.1.1.1: pyruvate decarboxylase (T81). EC 4.1.1.49: phosphoenolpyruvate carboxykinase (ATP). EC 4.1.2.13: fructose-bisphosphate aldolase, class I. EC 4.2.1.11: enolase. EC 5.1.3.15: glucose-6-phosphate 1-epimerase. EC 5.3.1.1: triosephosphate isomerase (TIM). EC 5.4.2.11: 2,3-bisphosphoglycerate-dependent phosphoglycerate mutase. EC 6.2.1.1: acetyl-CoA synthetase.

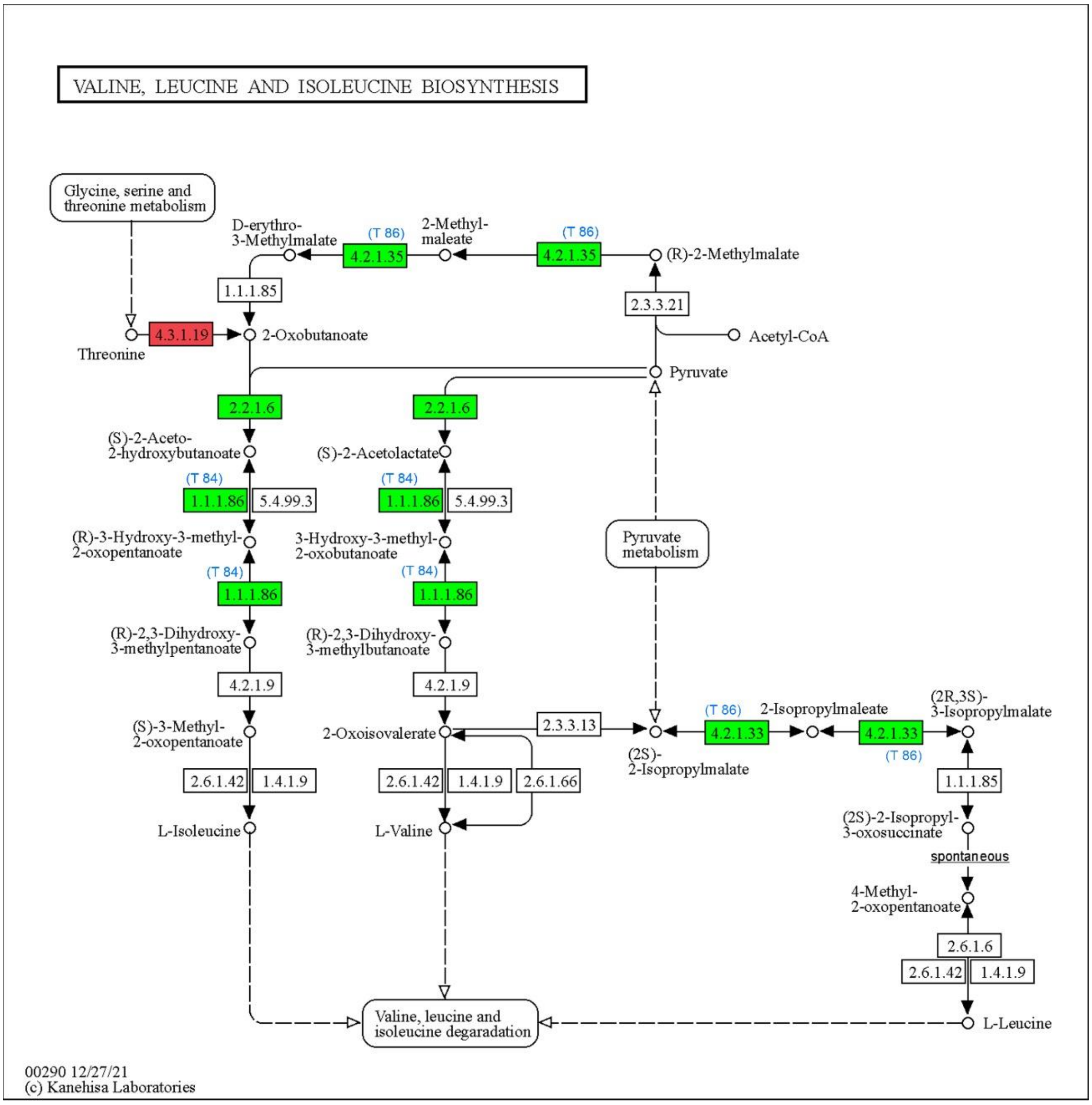

Supplementary Figure S14. KEGG diagram of genes associated with valine, leucine and isoleucine biosynthesis. The boxes represent enzymes and contain the respective EC numbers (Enzyme Commission number). The transcript which encodes the enzyme is indicated between blue brackets. Green and red indicate increased and decreased expression (FC ≥ 2 or FC ≤ -2, FDR < 0.001). EC 1.1.1.86: ketol-acid reductoisomerase (T84). EC 2.2.1.6: acetolactate synthase I/II/III large subunit. EC 4.2.1.33: 3-isopropylmalate dehydratase. EC 4.2.1.35: 3-isopropylmalate/(R)-2-methylmalate dehydratase large subunit (T86). EC 4.3.1.19: L-serine/L-threonine ammonia-lyase.

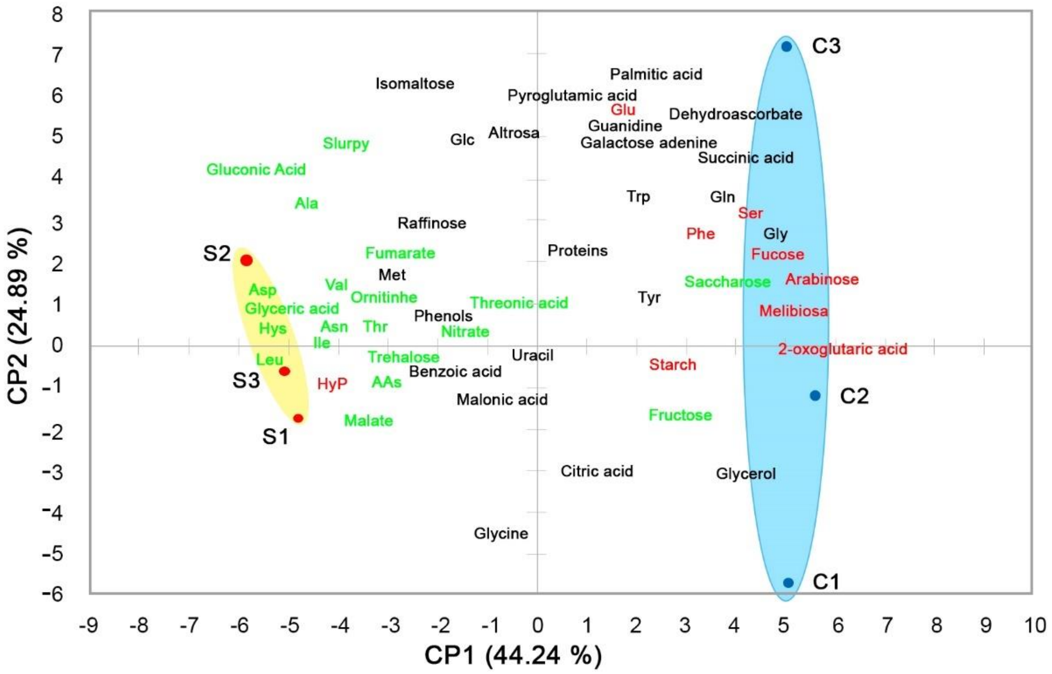

Supplementary Figure S15. Root metabolic profiles principal component analysis from non-stressed (C1, C2 and C3) and stressed (S1, S2 and S3) plants. PC1, principal component 1; PC2, principal component 2. Green, red, or black compounds indicate an increase, diminution, or no significant changes in their concentration due to water stress.

Supplementary Table S1. Quality assessment determination of de novo transcriptome assemblies using BLASTn. Gene **s**equence description.

Sequence stored in GenBank				Assembled transcript		Similarity		
Accession number	Annotation	CDS	Length	ID	Length	Identities/ Alignment length	Identity % of alignment	E-value
JX131367.1	aspartate aminotransferase	partial	1,212	c57434_g9_i2	742	689/691	99	0.0
JX271039.1	metallothionein type 2	complete	525	c54201_g3_i2	875	513/514	99	0.0
JX261967.1	succinate dehydrogenase flavoprotein subunit	partial	391	c58482_g2_i3	2386	145/175	82	9e-023
KC491213.1	protein phosphatase 2C	complete	1329	c55988_g1_i6	1552	1317/1319	99	0.0
KC505154.1	hypothetical protein B1	complete	1286	c53427_g2_i1	1238	1169/1171	99	0.0
KC505153.1	beta-tubulin	complete	1618	c58434_g4_i1	1108	1056/1059	99	0.0
KT428870.1	E3 transcription activator GLK1-like	partial	1449	c18378_g1_i1	941	877/877	100	0.0
KT428869.1	replication protein A 32 kDa subunit B-like	partial	993	c45930_g1_i1	1418	992/992	100	0.0
KT428868.1	basic 7S globulin-like	partial	1415	c48115_g1_i1	1561	1401/1401	100	0.0
KT428850.1	protein TIME FOR COFFEE-like	partial	1166	c57476_g1_i3	4097	817/818	99	0.0
KT428841.1	stellacyanin	partial	1420	c51285_g2_i1	1369	1362/1363	99	0.0
KT428840.1	chorismate mutase-like	partial	1375	c58864_g3_i1	1302	1300/1300	100	0.0
KT441133.1	photosystem I P700 chlorophyll a apoprotein A1	partial	1275	c56555_g3_i2	1089	1046/1046	100	0.0
KT441134.1	RuBisCO activase beta isoform	complete	2033	c45472_g1_i1	1638	1381/1387	99	0.0
KT441135.1	RuBisCO activase alpha isoform	complete	1998	c45472_g1_i1	1638	1632/1638	99	0.0
KT441132.1	threonyl-tRNA synthetase	partial	2502	c59453_g2_i10	2840	2378/2381	99	0.0
KT441131.1	calcium-binding protein CML29-like	partial	795	c39641_g1_i1	712	712/712	100	0.0
KT441130.1	ubiquitin conjugation factor E4-like	complete	3885	c60117_g2_i4	3339	3305/3310	99	0.0
KT441129.1	E3 ubiquitin-protein ligase RING1-like	partial	1436	c55858_g5_i4	1758	966/967	99	0.0
KT441128.1	zinc/iron transporter	complete	1424	c55380_g1_i1	1731	1316/1382	95	0.0
KT441126.1	walls are thin 1	complete	1885	c60152_g4_i4	1379	1236/1236	100	0.0
KU886196.1	polyubiquitin	partial	1160	c58000_g2_i1	334	292/300	97	e-149
KU886197.1	elongation factor 1-alpha	complete	1584	c59643_g1_i4	1833	1042/1147	90	0.0

Table S1 (*continued*)

**Sequence stored in GenBank**				**Assembled transcript**		**Similarity**		
Accession number	Annotation	CDS	Length	ID	Length	Identities/ Alignment length	Identity % of alignment	E-value
KU886198.1	actin	partial	373	c55401_g4_i6	438	370/370	100	0.0
KU886199.1	tubulin alpha chain	complete	1417	c58058_g1_i4	3080	1413/1414	99	0.0
KU886200.1	cyclophilin 38	complete	1491	c54764_g2_i2	1952	1489/1491	99	0.0
KU886201.1	RNA polymerase-associated protein RTF1-like protein	partial	488	c59207_g7_i1	1505	383/388	98	0.0
KU886202.1	4-hydroxy-3-methylbut-2-en-1-yl diphosphate synthase	complete	2338	c55168_g1_i6	2739	2335/2338	99	0.0
KU886203.1	vacuolar protein sorting-associated protein	complete	3094	c54068_g5_i5	3469	3091/3094	99	0.0
KT441125.1	isopiperitenol/carveol dehydrogenase	partial	995	c59308_g1_i1	1136	990/995	99	0.0
KT441127.1	chromatin modification protein MEAF6-like	partial	570	c59306_g4_i10	1125	546/546	100	0.0
KT441136.1	EST clone F15	-	2152	c55526_g1_i3	1419	923/1025	90	0.0
KT441137.1	EST clone G12	-	1046	c36273_g1_i1	1082	1046/1046	100	0.0
KT441138.1	EST clone G23	-	999	c12028_g1_i1	1071	999/999	100	0.0
KT428842.1	uncharacterized protein isolate G3	-	521	c51358_g1_i2	680	516/517	99	0.0
KT428843.1	uncharacterized protein isolate G5	-	326	c50167_g1_i3	1150	24/24	100	2e-004
KT428844.1	uncharacterized protein isolate G10	-	1733	c56688_g3_i1	1607	1598/1603	99	0.0
KT428845.1	uncharacterized protein isolate G11	-	799	c39061_g1_i1	1114	785/787	99	0.0
KT428846.1	uncharacterized protein isolate G14	-	1171	c42648_g1_i1	1828	1171/1171	100	0.0
KT428847.1	EST isolate G1	-	339	c59544_g3_i4	2639	57/60	97	1e-018
KT428848.1	EST isolate G2	-	1081	c46682_g1_i1	652	651/652	99	0.0
KT428849.1	EST isolate G4	-	549	c34389_g1_i1	854	549/549	100	0.0
KT428851.1	glycine-rich cell wall structural protein-like	partial	741	c57917_g2_i1	1775	738/741	99	0.0
KT428852.1	ubiquitin-conjugating enzyme E2 35-like	partial	422	c84994_g1_i1	483	422/422	100	0.0
KT428853.1	EG45-like domain containing protein-like	partial	812	c51136_g2_i1	762	762/763	99	0.0
KT428854.1	protein of unknown function (DUF3353)	partial	1263	c46818_g1_i1	1093	1092/1093	99	0.0
KT428855.1	uncharacterized protein	partial	630	c14454_g1_i1	529	401/402	99	0.0

Table S1 (*continued*)

**Sequence stored in GenBank**				**Assembled transcript**		**Similarity**		
Accession number	Annotation	CDS	Length	ID	Length	Identities/ Alignment length	Identity % of alignment	E-value
KT428856.1	uncharacterized protein	partial	1190	c59382_g2_i2	2425	1190/1190	100	0.0
KT428857.1	uncharacterized protein	partial	553	c47531_g1_i1	558	487/495	98	0.0
KT428858.1	uncharacterized protein	partial	555	c58262_g1_i2	652	477/533	89	e-173
KT428859.1	uncharacterized protein	partial	1183	c42903_g1_i1	1192	1180/1183	99	0.0
KT428860.1	uncharacterized protein	partial	516	c58248_g8_i6	1603	371/430	86	e-105
KT428861.1	uncharacterized protein	partial	582	c38927_g1_i1	540	523/523	100	0.0
KT428862.1	uncharacterized protein	partial	779	c59446_g1_i1	1278	779/779	100	0.0
KT428863.1	EST isolate B15	partial	539	c17933_g1_i1	1826	539/539	100	0.0
KT428864.1	EST isolate C8	partial	753	c48205_g2_i1	1947	753/753	100	0.0
KT428865.1	EST isolate F14	partial	499	c51786_g1_i3	1499	498/498	100	0.0
KT428866.1	EST isolate F5	partial	383	c58520_g6_i4	563	383/383	100	0.0
KT428867.1	heat shock 70 kDa protein 8-like	partial	1854	c44151_g1_i1	2180	1854/1854	100	0.0
KT428871.1	uncharacterized protein	partial	807	c21930_g1_i1	1293	807/807	100	0.0
KT428872.1	uncharacterized protein	partial	314	c150144_g1_i1	298	296/296	100	e-166
KT428874.1	EST isolate G8	-	625	c43795_g1_i1	575	571/575	99	0.0

Supplementary Table S2. Transcript level quantification by real-time qPCR. The values are the mean of three plants and are expressed as the x-fold change respectively to control. Significant differences were determined by t-test (P <0.05).

		**Treatment**		
		**Control**	**Stress**	
**Gene**	**Primer name**	**Mean ± Std. Error of Mean**	**Mean ± SEM**	**t-test**
RS-1 protein (RSI-1)	RSI	1 ± 0,0866025	0,000190067 ± 0,000063509	***
Polyphenol oxidase (PPO)	PPO	1 ± 0,132791	0,00262177 ± 0,00016667	**
Raffinose synthase (RafS)	RAF	1 ± 0,11547	0,692555 ± 0,230940	NS
Alternative oxidase (AOX)	AOX	1 ± 0,057735	7,05184 ± 0,23094	****
Trehalose phosphate synthase (TPS)	TREH	1 ± 0,057735	2,39994 ± 0,17321	**
1-aminocyclopropane-1-carboxylate oxidase (ACC)	ACC	1 ± 0,0173205	1,25701 ± 0,11547	NS
Galactinol synthase (GolS)	GOL	1 ± 0,0866025	16,0111 ± 0,2887	****
1,4-alpha-glucan branching enzyme *(GBE1)*	BRAN	1 ± 0,0635085	132,5140 ± 0,57735	****
Uncharacterized transcript (c58016_g2_i2)	UNTR	1 ± 0,288675	1,14155 ± 0,17321	NS

Supplementary Table S3. Functions enriched in up-regulated transcripts according to Fisher's Exact Test. Terms were classified according to the GO category and ordered from lowest to highest FDR.

GO category	GO ID	GO name	FDR	P-Value
Biological Process	GO:0030245	cellulose catabolic process	4,71E-14	3,61E-16
Biological Process	GO:0039694	viral RNA genome replication	4,27E-13	3,68E-15
Biological Process	GO:0009414	response to water deprivation	7,07E-13	6,17E-15
Biological Process	GO:0009738	abscisic acid-activated signalling pathway	7,36E-11	7,47E-13
Biological Process	GO:0009408	response to heat	2,00E-10	2,08E-12
Biological Process	GO:0010200	response to chitin	3,68E-09	4,26E-11
Biological Process	GO:0006414	translational elongation	3,77E-09	4,38E-11
Biological Process	GO:0009651	response to salt stress	1,18E-08	1,42E-10
Biological Process	GO:0009739	response to gibberellin	1,36E-07	1,73E-09
Biological Process	GO:0008643	carbohydrate transport	5,55E-07	7,51E-09
Biological Process	GO:0009644	response to the high-light intensity	1,19E-06	1,70E-08
Biological Process	GO:0009409	response to cold	4,79E-06	7,22E-08
Biological Process	GO:0019725	cellular homeostasis	6,15E-06	9,38E-08
Biological Process	GO:0009753	response to jasmonic acid	1,54E-05	2,42E-07
Biological Process	GO:0009873	ethylene-activated signalling pathway	2,72E-05	4,40E-07
Biological Process	GO:0098542	defence response to other organisms	4,09E-05	6,88E-07
Biological Process	GO:1901615	organic hydroxy compound metabolic process	6,62E-05	1,14E-06
Biological Process	GO:0042542	response to hydrogen peroxide	6,74E-05	1,17E-06
Biological Process	GO:0045727	positive regulation of translation	6,74E-05	1,17E-06
Biological Process	GO:0016043	cellular component organization	6,74E-05	1,18E-06
Biological Process	GO:0006812	cation transport	7,61E-05	1,35E-06
Biological Process	GO:0009228	thiamine biosynthetic process	1,05E-04	1,90E-06
Biological Process	GO:2000242	negative regulation of the reproductive process	1,14E-04	2,07E-06
Biological Process	GO:0048573	photoperiodism, flowering	1,35E-04	2,49E-06
Biological Process	GO:0009405	pathogenesis	1,70E-04	3,18E-06
Biological Process	GO:0010360	negative regulation of anion channel activity	1,91E-04	3,60E-06
Biological Process	GO:0045892	negative regulation of transcription, DNA-templated	2,08E-04	3,95E-06
Biological Process	GO:0048580	regulation of post-embryonic development	2,19E-04	4,22E-06
Biological Process	GO:0016311	dephosphorylation	2,21E-04	4,26E-06
Biological Process	GO:0009733	response to auxin	2,46E-04	4,79E-06
Biological Process	GO:0010224	response to UV-B	2,77E-04	5,44E-06
Biological Process	GO:0009908	flower development	2,97E-04	5,86E-06
Biological Process	GO:0006081	cellular aldehyde metabolic process	3,11E-04	6,15E-06
Biological Process	GO:0055065	metal ion homeostasis	4,06E-04	8,09E-06
Biological Process	GO:0045893	positive regulation of transcription, DNA-templated	4,18E-04	8,46E-06
Biological Process	GO:0048838	release of seed from dormancy	5,12E-04	1,05E-05
Biological Process	GO:0035690	cellular response to drug	5,21E-04	1,08E-05
Biological Process	GO:0045493	xylan catabolic process	5,24E-04	1,09E-05
Biological Process	GO:0051239	regulation of multicellular organismal process	6,41E-04	1,37E-05
Biological Process	GO:0007623	circadian rhythm	8,94E-04	1,93E-05

Biological Process	GO:0031668	cellular response to extracellular stimulus	9,34E-04	2,04E-05
Biological Process	GO:0043620	regulation of DNA-templated transcription in response to stress	9,34E-04	2,04E-05
Biological Process	GO:0044092	negative regulation of molecular function	9,71E-04	2,13E-05
Biological Process	GO:0061013	regulation of mRNA catabolic process	9,83E-04	2,16E-05
Molecular function	GO:0003735	structural constituent of ribosome	1,13E-21	6,04E-24
Molecular function	GO:0003723	RNA binding	2,12E-16	1,41E-18
Molecular function	GO:0008810	cellulase activity	3,12E-12	2,81E-14
Molecular function	GO:0030248	cellulose binding	6,77E-11	6,79E-13
Molecular function	GO:0003968	RNA-directed 5'-3' RNA polymerase activity	1,14E-09	1,26E-11
Molecular function	GO:0004722	protein serine/threonine phosphatase activity	5,91E-08	7,29E-10
Molecular function	GO:0008174	mRNA methyltransferase activity	7,04E-08	8,74E-10
Molecular function	GO:0005515	protein binding	8,20E-08	1,03E-09
Molecular function	GO:0140096	catalytic activity, acting on a protein	3,09E-07	4,00E-09
Molecular function	GO:0000166	nucleotide binding	3,21E-07	4,19E-09
Molecular function	GO:0000976	transcription regulatory region sequence-specific DNA binding	5,72E-07	7,78E-09
Molecular function	GO:0003682	chromatin binding	3,16E-06	4,67E-08
Molecular function	GO:0043168	anion binding	4,08E-06	6,10E-08
Molecular function	GO:0015293	symporter activity	2,80E-05	4,57E-07
Molecular function	GO:0050662	obsolete coenzyme binding	5,55E-05	9,53E-07
Molecular function	GO:0015078	proton transmembrane transporter activity	7,29E-05	1,28E-06
Molecular function	GO:0016740	transferase activity	1,25E-04	2,29E-06
Molecular function	GO:0016829	lyase activity	2,36E-04	4,57E-06
Molecular function	GO:0005216	ion channel activity	2,69E-04	5,26E-06
Molecular function	GO:0016530	metallochaperone activity	3,96E-04	7,87E-06
Molecular function	GO:0046943	carboxylic acid transmembrane transporter activity	4,15E-04	8,31E-06
Molecular function	GO:0003746	translation elongation factor activity	4,33E-04	8,80E-06
Molecular function	GO:0016788	hydrolase activity, acting on ester bonds	4,78E-04	9,73E-06
Molecular function	GO:0030570	pectate lyase activity	5,63E-04	1,18E-05
Molecular function	GO:0000982	DNA-binding transcription factor activity, RNA polymerase II-specific	5,80E-04	1,22E-05
Molecular function	GO:0016614	oxidoreductase activity, acting on CH-OH donor group	8,22E-04	1,77E-05
Molecular function	GO:0008270	zinc ion binding	9,11E-04	1,97E-05
Molecular function	GO:0015035	protein disulfide oxidoreductase activity	9,30E-04	2,02E-05
Cellular Component	GO:0005634	nucleus	2,18E-43	5,07E-46
Cellular Component	GO:0005576	extracellular region	1,46E-25	6,23E-28
Cellular Component	GO:0019028	viral capsid	4,10E-10	4,38E-12
Cellular Component	GO:0005829	cytosol	2,73E-09	3,13E-11
Cellular Component	GO:0031225	anchored component of membrane	1,69E-07	2,16E-09
Cellular Component	GO:0031969	chloroplast membrane	1,56E-06	2,23E-08
Cellular Component	GO:0009570	chloroplast stroma	1,25E-05	1,96E-07
Cellular Component	GO:0044391	ribosomal subunit	2,80E-05	4,55E-07
Cellular Component	GO:0005887	integral component of the plasma membrane	4,16E-05	7,02E-07
Cellular Component	GO:0005773	vacuole	2,13E-04	4,07E-06
Cellular Component	GO:0005777	peroxisome	6,04E-04	1,28E-05

Cellular Component	GO:0009535	chloroplast thylakoid membrane	6,20E-04	1,32E-05

Supplementary Table S4. Functions enriched in down-regulated transcripts according to Fisher's Exact Test. Terms were classified according to the GO category and ordered from lowest to highest FDR.

GO category	GO ID	GO name	FDR	P-Value
Biological Processes	GO:0009834	plant-type secondary cell wall biogenesis	1,36E-27	1,78E-29
Biological Processes	GO:0007018	microtubule-based movement	3,69E-27	4,95E-29
Biological Processes	GO:0009809	lignin biosynthetic process	9,34E-27	1,30E-28
Biological Processes	GO:0030244	cellulose biosynthetic process	5,89E-20	1,02E-21
Biological Processes	GO:0009409	response to cold	6,87E-18	1,26E-19
Biological Processes	GO:0016042	lipid catabolic process	1,70E-17	3,20E-19
Biological Processes	GO:0042744	hydrogen peroxide catabolic process	5,28E-16	1,07E-17
Biological Processes	GO:0010067	procambium histogenesis	2,38E-15	5,01E-17
Biological Processes	GO:0045490	pectin catabolic process	1,27E-14	2,78E-16
Biological Processes	GO:0080167	response to karrikin	3,55E-13	8,22E-15
Biological Processes	GO:0009414	response to water deprivation	3,78E-13	8,79E-15
Biological Processes	GO:0046274	lignin catabolic process	5,38E-13	1,26E-14
Biological Processes	GO:0050832	defence response to fungus	4,74E-10	1,35E-11
Biological Processes	GO:0030001	metal ion transport	4,79E-10	1,37E-11
Biological Processes	GO:0042742	defence response to bacterium	6,35E-10	1,83E-11
Biological Processes	GO:0009686	gibberellin biosynthetic process	6,48E-10	1,88E-11
Biological Processes	GO:0009873	ethylene-activated signalling pathway	8,12E-10	2,36E-11
Biological Processes	GO:0006833	water transport	1,02E-09	3,00E-11
Biological Processes	GO:0009835	fruit ripening	2,24E-09	6,64E-11
Biological Processes	GO:0009742	brassinosteroid-mediated signalling pathway	2,69E-09	8,04E-11
Biological Processes	GO:0046686	response to cadmium ion	3,88E-09	1,18E-10
Biological Processes	GO:0006334	nucleosome assembly	4,55E-09	1,39E-10
Biological Processes	GO:0009624	response to nematode	4,88E-09	1,49E-10
Biological Processes	GO:0009820	alkaloid metabolic process	5,97E-09	1,84E-10
Biological Processes	GO:0016567	protein ubiquitination	8,65E-09	2,69E-10
Biological Processes	GO:0007623	circadian rhythm	3,69E-08	1,20E-09
Biological Processes	GO:0042128	nitrate assimilation	1,14E-07	3,84E-09
Biological Processes	GO:0006869	lipid transport	1,16E-07	3,96E-09
Biological Processes	GO:0009611	response to wounding	1,57E-07	5,44E-09
Biological Processes	GO:0019761	glucosinolate biosynthetic process	2,39E-07	8,46E-09
Biological Processes	GO:0045489	pectin biosynthetic process	3,07E-07	1,09E-08
Biological Processes	GO:0042538	hyperosmotic salinity response	3,54E-07	1,27E-08
Biological Processes	GO:0009740	gibberellic acid mediated signalling pathway	4,72E-07	1,71E-08
Biological Processes	GO:0006014	D-ribose metabolic process	5,34E-07	1,95E-08
Biological Processes	GO:0009718	anthocyanin-containing compound biosynthetic process	6,14E-07	2,27E-08
Biological Processes	GO:0009877	nodulation	7,70E-07	2,90E-08
Biological Processes	GO:0010305	leaf vascular tissue pattern formation	7,70E-07	2,90E-08
Biological Processes	GO:0009626	plant-type hypersensitive response	9,29E-07	3,53E-08
Biological Processes	GO:0000079	regulation of cyclin-dependent protein serine/threonine kinase activity	1,72E-06	6,75E-08

Biological Processes	GO:0006949	syncytium formation	1,77E-06	6,94E-08
Biological Processes	GO:0009833	plant-type primary cell wall biogenesis	1,83E-06	7,20E-08
Biological Processes	GO:0046777	protein autophosphorylation	2,07E-06	8,17E-08
Biological Processes	GO:0019722	calcium-mediated signalling	2,07E-06	8,21E-08
Biological Processes	GO:0010417	glucuronoxylan biosynthetic process	2,81E-06	1,12E-07
Biological Processes	GO:0010588	cotyledon vascular tissue pattern formation	4,16E-06	1,70E-07
Biological Processes	GO:0001887	selenium compound metabolic process	5,09E-06	2,09E-07
Biological Processes	GO:0035865	cellular response to potassium ion	5,09E-06	2,09E-07
Biological Processes	GO:0046855	inositol phosphate dephosphorylation	6,08E-06	2,51E-07
Biological Processes	GO:0009738	abscisic acid-activated signalling pathway	6,94E-06	2,88E-07
Biological Processes	GO:0009944	polarity specification of the adaxial/abaxial axis	7,71E-06	3,22E-07
Biological Processes	GO:0071241	cellular response to inorganic substance	8,43E-06	3,55E-07
Biological Processes	GO:0009860	pollen tube growth	8,48E-06	3,58E-07
Biological Processes	GO:0031408	oxylipin biosynthetic process	9,75E-06	4,16E-07
Biological Processes	GO:1901959	positive regulation of cutin biosynthetic process	9,97E-06	4,26E-07
Biological Processes	GO:0055088	lipid homeostasis	1,17E-05	5,06E-07
Biological Processes	GO:0032957	inositol trisphosphate metabolic process	1,19E-05	5,18E-07
Biological Processes	GO:0010073	meristem maintenance	1,74E-05	7,70E-07
Biological Processes	GO:0048523	negative regulation of cellular process	1,77E-05	7,87E-07
Biological Processes	GO:0006032	chitin catabolic process	2,20E-05	9,89E-07
Biological Processes	GO:0010091	trichome branching	2,22E-05	1,00E-06
Biological Processes	GO:0009308	amine metabolic process	2,24E-05	1,01E-06
Biological Processes	GO:0008283	cell population proliferation	2,38E-05	1,08E-06
Biological Processes	GO:0045089	positive regulation of innate immune response	2,39E-05	1,08E-06
Biological Processes	GO:0016036	cellular response to phosphate starvation	2,42E-05	1,10E-06
Biological Processes	GO:0033383	geranyl diphosphate metabolic process	2,49E-05	1,13E-06
Biological Processes	GO:0045893	positive regulation of transcription, DNA-templated	2,62E-05	1,20E-06
Biological Processes	GO:0010187	negative regulation of seed germination	2,74E-05	1,26E-06
Biological Processes	GO:0009411	response to UV	2,88E-05	1,33E-06
Biological Processes	GO:0048767	root hair elongation	2,90E-05	1,34E-06
Biological Processes	GO:0000070	mitotic sister chromatid segregation	3,10E-05	1,43E-06
Biological Processes	GO:0009786	regulation of asymmetric cell division	3,12E-05	1,45E-06
Biological Processes	GO:0010023	proanthocyanidin biosynthetic process	3,93E-05	1,84E-06
Biological Processes	GO:0009744	response to sucrose	4,18E-05	1,96E-06
Biological Processes	GO:0048638	regulation of developmental growth	4,81E-05	2,29E-06
Biological Processes	GO:0051607	defense response to virus	4,84E-05	2,31E-06
Biological Processes	GO:0052546	cell wall pectin metabolic process	5,11E-05	2,44E-06
Biological Processes	GO:0006270	DNA replication initiation	5,46E-05	2,62E-06
Biological Processes	GO:0009119	ribonucleoside metabolic process	5,74E-05	2,76E-06
Biological Processes	GO:0042278	purine nucleoside metabolic process	6,63E-05	3,21E-06
Biological Processes	GO:0009234	menaquinone biosynthetic process	6,71E-05	3,27E-06
Biological Processes	GO:0010260	animal organ senescence	6,71E-05	3,27E-06
Biological Processes	GO:0010215	cellulose microfibril organization	7,57E-05	3,74E-06
Biological Processes	GO:0006268	DNA unwinding involved in DNA replication	7,59E-05	3,75E-06

Biological Processes	GO:0010228	vegetative to the reproductive phase transition of meristem	8,18E-05	4,05E-06
Biological Processes	GO:0009862	systemic acquired resistance, salicylic acid-mediated signalling pathway	8,41E-05	4,18E-06
Biological Processes	GO:0016998	cell wall macromolecule catabolic process	8,66E-05	4,31E-06
Biological Processes	GO:1901607	alpha-amino acid biosynthetic process	8,76E-05	4,36E-06
Biological Processes	GO:0048232	male gamete generation	8,83E-05	4,40E-06
Biological Processes	GO:0009117	nucleotide metabolic process	9,52E-05	4,77E-06
Biological Processes	GO:0010411	xyloglucan metabolic process	1,02E-04	5,13E-06
Biological Processes	GO:0090333	regulation of stomatal closure	1,03E-04	5,17E-06
Biological Processes	GO:0010167	response to nitrate	1,03E-04	5,17E-06
Biological Processes	GO:0045168	cell-cell signalling involved in cell fate commitment	1,07E-04	5,38E-06
Biological Processes	GO:0000724	double-strand break repair via homologous recombination	1,11E-04	5,60E-06
Biological Processes	GO:0010646	regulation of cell communication	1,16E-04	5,89E-06
Biological Processes	GO:0010231	maintenance of seed dormancy	1,20E-04	6,08E-06
Biological Processes	GO:0048653	anther development	1,23E-04	6,27E-06
Biological Processes	GO:0009735	response to cytokinin	1,27E-04	6,48E-06
Biological Processes	GO:0010315	auxin efflux	1,51E-04	7,78E-06
Biological Processes	GO:0048016	inositol phosphate-mediated signalling	1,57E-04	8,12E-06
Biological Processes	GO:0009658	chloroplast organization	1,72E-04	8,99E-06
Biological Processes	GO:0019755	one-carbon compound transport	1,80E-04	9,50E-06
Biological Processes	GO:0042547	cell wall modification involved in multidimensional cell growth	1,80E-04	9,51E-06
Biological Processes	GO:0010069	zygote asymmetric cytokinesis in the embryo sac	1,80E-04	9,51E-06
Biological Processes	GO:0090407	organophosphate biosynthetic process	1,91E-04	1,01E-05
Biological Processes	GO:0009299	mRNA transcription	1,94E-04	1,02E-05
Biological Processes	GO:0009108	obsolete coenzyme biosynthetic process	1,95E-04	1,03E-05
Biological Processes	GO:0010286	heat acclimation	2,00E-04	1,06E-05
Biological Processes	GO:0016104	triterpenoid biosynthetic process	2,00E-04	1,06E-05
Biological Processes	GO:0010227	floral organ abscission	2,01E-04	1,07E-05
Biological Processes	GO:0006695	cholesterol biosynthetic process	2,04E-04	1,09E-05
Biological Processes	GO:0048359	mucilage metabolic process involved in seed coat development	2,04E-04	1,09E-05
Biological Processes	GO:0006636	unsaturated fatty acid biosynthetic process	2,06E-04	1,11E-05
Biological Processes	GO:0009640	photomorphogenesis	2,28E-04	1,23E-05
Biological Processes	GO:0010114	response to red light	2,32E-04	1,26E-05
Biological Processes	GO:0010311	lateral root formation	2,56E-04	1,39E-05
Biological Processes	GO:0080001	mucilage extrusion from the seed coat	2,56E-04	1,40E-05
Biological Processes	GO:0010374	stomatal complex development	2,61E-04	1,42E-05
Biological Processes	GO:0071417	cellular response to organonitrogen compound	2,82E-04	1,54E-05
Biological Processes	GO:0007166	cell surface receptor signalling pathway	2,83E-04	1,55E-05
Biological Processes	GO:0042814	monopolar cell growth	2,87E-04	1,58E-05
Biological Processes	GO:0046839	phospholipid dephosphorylation	2,98E-04	1,64E-05
Biological Processes	GO:0010025	wax biosynthetic process	3,04E-04	1,68E-05
Biological Processes	GO:0010089	xylem development	3,25E-04	1,80E-05
Biological Processes	GO:0046463	acylglycerol biosynthetic process	3,41E-04	1,89E-05

Biological Processes	GO:0010150	leaf senescence	3,42E-04	1,90E-05
Biological Processes	GO:0044770	cell cycle phase transition	3,47E-04	1,94E-05
Biological Processes	GO:0031669	cellular response to nutrient levels	3,48E-04	1,94E-05
Biological Processes	GO:0006767	water-soluble vitamin metabolic process	3,91E-04	2,19E-05
Biological Processes	GO:0016131	brassinosteroid metabolic process	4,08E-04	2,30E-05
Biological Processes	GO:0009958	positive gravitropism	4,08E-04	2,30E-05
Biological Processes	GO:0007155	cell adhesion	4,43E-04	2,52E-05
Biological Processes	GO:0010540	basipetal auxin transport	4,48E-04	2,54E-05
Biological Processes	GO:0098662	inorganic cation transmembrane transport	4,66E-04	2,65E-05
Biological Processes	GO:0010358	leaf shaping	4,84E-04	2,77E-05
Biological Processes	GO:0046168	glycerol-3-phosphate catabolic process	4,88E-04	2,80E-05
Biological Processes	GO:0070085	glycosylation	5,05E-04	2,92E-05
Biological Processes	GO:0042542	response to hydrogen peroxide	5,53E-04	3,21E-05
Biological Processes	GO:0006304	DNA modification	5,62E-04	3,26E-05
Biological Processes	GO:0022412	cellular process involved in reproduction in multicellular organisms	5,79E-04	3,37E-05
Biological Processes	GO:0009827	plant-type cell wall modification	5,91E-04	3,45E-05
Biological Processes	GO:0000919	cell plate assembly	6,15E-04	3,60E-05
Biological Processes	GO:0009294	DNA mediated transformation	6,21E-04	3,64E-05
Biological Processes	GO:0034404	nucleobase-containing small molecule biosynthetic process	6,28E-04	3,69E-05
Biological Processes	GO:0046942	carboxylic acid transport	6,61E-04	3,90E-05
Biological Processes	GO:0009867	jasmonic acid mediated signalling pathway	6,94E-04	4,09E-05
Biological Processes	GO:0023051	regulation of signalling	7,66E-04	4,53E-05
Biological Processes	GO:0009556	microsporogenesis	7,66E-04	4,53E-05
Biological Processes	GO:0006714	sesquiterpenoid metabolic process	7,79E-04	4,61E-05
Biological Processes	GO:0000075	cell cycle checkpoint	8,08E-04	4,80E-05
Biological Processes	GO:0016099	monoterpenoid biosynthetic process	8,17E-04	4,88E-05
Biological Processes	GO:0015706	nitrate transport	8,17E-04	4,88E-05
Biological Processes	GO:0055076	transition metal ion homeostasis	8,18E-04	4,89E-05
Biological Processes	GO:0033358	UDP-L-arabinose biosynthetic process	8,65E-04	5,24E-05
Biological Processes	GO:0043693	monoterpene biosynthetic process	8,65E-04	5,24E-05
Biological Processes	GO:0010425	DNA methylation on cytosine within a CNG sequence	8,65E-04	5,24E-05
Biological Processes	GO:0009612	response to mechanical stimulus	8,87E-04	5,39E-05
Biological Processes	GO:0016571	histone methylation	8,89E-04	5,40E-05
Biological Processes	GO:0010043	response to zinc ion	8,91E-04	5,42E-05
Biological Processes	GO:0000226	microtubule cytoskeleton organization	9,19E-04	5,60E-05
Biological Processes	GO:0042180	cellular ketone metabolic process	9,87E-04	6,10E-05
Biological Processes	GO:0009110	vitamin biosynthetic process	9,98E-04	6,17E-05
Molecular function	GO:0005524	ATP binding	6,46E-73	2,72E-75
Molecular function	GO:0003700	DNA-binding transcription factor activity	6,90E-67	3,40E-69
Molecular function	GO:0020037	heme binding	1,00E-59	6,00E-62
Molecular function	GO:0005506	iron ion binding	1,25E-49	9,36E-52
Molecular function	GO:0005507	copper ion binding	2,06E-32	2,21E-34
Molecular function	GO:0045735	nutrient reservoir activity	1,49E-20	2,54E-22

Molecular function	GO:0043565	sequence-specific DNA binding	4,57E-19	8,11E-21
Molecular function	GO:0016760	cellulose synthase (UDP-forming) activity	7,06E-18	1,30E-19
Molecular function	GO:0004185	serine-type carboxypeptidase activity	5,22E-17	1,00E-18
Molecular function	GO:0030599	pectinesterase activity	1,03E-16	1,97E-18
Molecular function	GO:0008762	UDP-N-acetylmuramate dehydrogenase activity	1,15E-15	2,37E-17
Molecular function	GO:0004601	peroxidase activity	3,07E-15	6,48E-17
Molecular function	GO:0003682	chromatin binding	3,07E-15	6,50E-17
Molecular function	GO:0008017	microtubule binding	1,76E-14	3,90E-16
Molecular function	GO:0045330	aspartyl esterase activity	1,68E-12	4,05E-14
Molecular function	GO:0052716	hydroquinone:oxygen oxidoreductase activity	2,21E-12	5,36E-14
Molecular function	GO:0042973	glucan endo-1,3-beta-D-glucosidase activity	4,25E-12	1,04E-13
Molecular function	GO:0050660	flavin adenine dinucleotide binding	8,53E-12	2,13E-13
Molecular function	GO:0015250	water channel activity	2,41E-11	6,23E-13
Molecular function	GO:0030145	manganese ion binding	3,27E-11	8,55E-13
Molecular function	GO:0051753	mannan synthase activity	9,77E-10	2,85E-11
Molecular function	GO:0015020	glucuronosyltransferase activity	2,45E-09	7,29E-11
Molecular function	GO:0050378	UDP-glucuronate 4-epimerase activity	2,49E-08	7,94E-10
Molecular function	GO:1990135	flavonoid sulfotransferase activity	2,49E-08	7,94E-10
Molecular function	GO:0033612	receptor serine/threonine kinase binding	9,69E-08	3,26E-09
Molecular function	GO:0031418	L-ascorbic acid binding	2,00E-07	7,01E-09
Molecular function	GO:0004565	beta-galactosidase activity	2,02E-07	7,09E-09
Molecular function	GO:0047262	polygalacturonate 4-alpha-galacturonosyltransferase activity	2,32E-07	8,16E-09
Molecular function	GO:0004747	ribokinase activity	5,34E-07	1,95E-08
Molecular function	GO:0008061	chitin binding	5,80E-07	2,13E-08
Molecular function	GO:0004806	triglyceride lipase activity	9,90E-07	3,77E-08
Molecular function	GO:0004675	transmembrane receptor protein serine/threonine kinase activity	1,02E-06	3,88E-08
Molecular function	GO:0015293	symporter activity	1,23E-06	4,76E-08
Molecular function	GO:0016799	hydrolase activity, hydrolyzing N-glycosyl compounds	1,43E-06	5,57E-08
Molecular function	GO:0042803	protein homodimerization activity	1,49E-06	5,82E-08
Molecular function	GO:0046029	mannitol dehydrogenase activity	2,99E-06	1,20E-07
Molecular function	GO:0003993	acid phosphatase activity	3,96E-06	1,61E-07
Molecular function	GO:0043531	ADP binding	4,16E-06	1,69E-07
Molecular function	GO:0005199	structural constituent of cell wall	4,64E-06	1,90E-07
Molecular function	GO:0004568	chitinase activity	4,68E-06	1,91E-07
Molecular function	GO:0016717	oxidoreductase activity, acting on paired donors, with oxidation of a pair of donors resulting in the reduction of molecular oxygen to two molecules of water	4,89E-06	2,00E-07
Molecular function	GO:0050590	desacetoxyvindoline 4-hydroxylase activity	6,73E-06	2,79E-07
Molecular function	GO:0003846	2-acylglycerol O-acyltransferase activity	7,71E-06	3,22E-07
Molecular function	GO:0009055	electron transfer activity	8,45E-06	3,57E-07
Molecular function	GO:0010333	terpene synthase activity	8,58E-06	3,64E-07
Molecular function	GO:0016765	transferase activity, transferring alkyl or aryl (other than methyl) groups	1,26E-05	5,48E-07
Molecular function	GO:0008395	steroid hydroxylase activity	1,95E-05	8,71E-07

Molecular function	GO:0003886	DNA (cytosine-5-)-methyltransferase activity	1,95E-05	8,71E-07
Molecular function	GO:0004842	ubiquitin-protein transferase activity	2,20E-05	9,86E-07
Molecular function	GO:0052659	inositol-1,3,4,5-tetrakisphosphate 5-phosphatase activity	2,49E-05	1,13E-06
Molecular function	GO:0008289	lipid binding	4,27E-05	2,01E-06
Molecular function	GO:0005179	hormone activity	4,41E-05	2,09E-06
Molecular function	GO:0015238	xenobiotic transmembrane transporter activity	4,64E-05	2,20E-06
Molecular function	GO:0080044	quercetin 7-O-glucosyltransferase activity	4,77E-05	2,27E-06
Molecular function	GO:0016762	xyloglucan:xyloglucosyl transferase activity	6,63E-05	3,21E-06
Molecular function	GO:0052658	inositol-1,4,5-trisphosphate 5-phosphatase activity	6,71E-05	3,27E-06
Molecular function	GO:0004630	phospholipase D activity	6,77E-05	3,30E-06
Molecular function	GO:0050636	vinorine synthase activity	7,09E-05	3,47E-06
Molecular function	GO:0005261	cation channel activity	7,41E-05	3,64E-06
Molecular function	GO:0004445	inositol-polyphosphate 5-phosphatase activity	9,00E-05	4,49E-06
Molecular function	GO:0008324	cation transmembrane transporter activity	9,41E-05	4,71E-06
Molecular function	GO:0016597	amino acid binding	1,38E-04	7,08E-06
Molecular function	GO:0033807	icosanoyl-CoA synthase activity	1,57E-04	8,14E-06
Molecular function	GO:0051777	ent-kaurenoate oxidase activity	1,57E-04	8,14E-06
Molecular function	GO:0090447	glycerol-3-phosphate 2-O-acyltransferase activity	1,57E-04	8,14E-06
Molecular function	GO:0047924	geraniol dehydrogenase activity	1,80E-04	9,51E-06
Molecular function	GO:0045544	gibberellin 20-oxidase activity	1,80E-04	9,51E-06
Molecular function	GO:0005200	structural constituent of cytoskeleton	1,87E-04	9,86E-06
Molecular function	GO:0033293	monocarboxylic acid binding	2,00E-04	1,06E-05
Molecular function	GO:0070290	N-acylphosphatidylethanolamine-specific phospholipase D activity	2,04E-04	1,09E-05
Molecular function	GO:0046873	metal ion transmembrane transporter activity	2,20E-04	1,18E-05
Molecular function	GO:0004506	squalene monooxygenase activity	2,87E-04	1,58E-05
Molecular function	GO:0008574	ATP-dependent microtubule motor activity, plus-end-directed	2,87E-04	1,58E-05
Molecular function	GO:0018685	alkane 1-monooxygenase activity	2,88E-04	1,59E-05
Molecular function	GO:0047372	acylglycerol lipase activity	3,62E-04	2,02E-05
Molecular function	GO:0030570	pectate lyase activity	3,89E-04	2,18E-05
Molecular function	GO:0010328	auxin influx transmembrane transporter activity	4,20E-04	2,38E-05
Molecular function	GO:0008131	primary amine oxidase activity	4,20E-04	2,38E-05
Molecular function	GO:0004650	polygalacturonase activity	4,42E-04	2,51E-05
Molecular function	GO:0008233	peptidase activity	4,73E-04	2,69E-05
Molecular function	GO:0008865	fructokinase activity	4,76E-04	2,72E-05
Molecular function	GO:0047364	desulfoglucosinolate sulfotransferase activity	4,84E-04	2,77E-05
Molecular function	GO:0004367	glycerol-3-phosphate dehydrogenase [NAD+] activity	4,88E-04	2,80E-05
Molecular function	GO:0015297	antiporter activity	4,88E-04	2,81E-05
Molecular function	GO:0004312	fatty acid synthase activity	5,35E-04	3,10E-05
Molecular function	GO:0010329	auxin efflux transmembrane transporter activity	6,21E-04	3,64E-05
Molecular function	GO:0050661	NADP binding	6,94E-04	4,10E-05
Molecular function	GO:0004310	farnesyl-diphosphate farnesyltransferase activity	7,84E-04	4,66E-05
Molecular function	GO:0051996	squalene synthase activity	7,84E-04	4,66E-05
Molecular function	GO:0015112	nitrate transmembrane transporter activity	8,17E-04	4,88E-05

Molecular function	GO:0016871	cycloartenol synthase activity	8,65E-04	5,24E-05
Molecular function	GO:0033809	anthocyanin 6''-O-malonyltransferase activity	8,65E-04	5,24E-05
Molecular function	GO:0050373	UDP-arabinose 4-epimerase activity	8,65E-04	5,24E-05
Molecular function	GO:0090353	polygalacturonase inhibitor activity	8,65E-04	5,24E-05
Molecular function	GO:0016407	acetyltransferase activity	9,80E-04	5,99E-05
Molecular function	GO:0003989	acetyl-CoA carboxylase activity	9,82E-04	6,06E-05
Molecular function	GO:0004144	diacylglycerol O-acyltransferase activity	9,82E-04	6,06E-05
Molecular function	GO:0004970	ionotropic glutamate receptor activity	9,82E-04	6,06E-05
Molecular function	GO:0005234	extracellularly glutamate-gated ion channel activity	9,82E-04	6,06E-05
Cellular Component	GO:0016021	integral component of the membrane	2,11E-234	1,25E-237
Cellular Component	GO:0048046	apoplast	4,07E-113	8,94E-116
Cellular Component	GO:0009506	plasmodesma	5,52E-70	2,56E-72
Cellular Component	GO:0005634	nucleus	1,67E-66	8,55E-69
Cellular Component	GO:0046658	anchored component of plasma membrane	1,71E-37	1,60E-39
Cellular Component	GO:0005871	kinesin complex	1,23E-30	1,50E-32
Cellular Component	GO:0009524	phragmoplast	5,80E-19	1,03E-20
Cellular Component	GO:0005802	trans-Golgi network	2,29E-17	4,37E-19
Cellular Component	GO:0005789	endoplasmic reticulum membrane	2,00E-15	4,18E-17
Cellular Component	GO:0005768	endosome	3,62E-13	8,40E-15
Cellular Component	GO:0005829	cytosol	4,97E-12	1,23E-13
Cellular Component	GO:0009570	chloroplast stroma	3,98E-11	1,05E-12
Cellular Component	GO:0000786	nucleosome	4,62E-10	1,31E-11
Cellular Component	GO:0031969	chloroplast membrane	2,59E-08	8,27E-10
Cellular Component	GO:0005615	extracellular space	6,26E-08	2,08E-09
Cellular Component	GO:0032580	Golgi cisterna membrane	1,67E-07	5,81E-09
Cellular Component	GO:0055028	cortical microtubule	1,86E-07	6,49E-09
Cellular Component	GO:0042807	central vacuole	5,11E-07	1,86E-08
Cellular Component	GO:0009705	plant-type vacuole membrane	3,73E-06	1,51E-07
Cellular Component	GO:0045178	basal part of the cell	4,41E-05	2,09E-06
Cellular Component	GO:0042555	MCM complex	7,09E-05	3,47E-06
Cellular Component	GO:0009531	secondary cell wall	7,57E-05	3,74E-06
Cellular Component	GO:0009504	cell plate	8,32E-05	4,13E-06
Cellular Component	GO:0000777	condensed chromosome kinetochore	2,03E-04	1,09E-05
Cellular Component	GO:0009579	thylakoid	5,69E-04	3,31E-05
Cellular Component	GO:0005876	spindle microtubule	6,21E-04	3,64E-05
Cellular Component	GO:0009317	acetyl-CoA carboxylase complex	7,84E-04	4,66E-05

Supplementary Table S5. Functional description of transcripts cited in the Results and Discussion sections.

ID	Transcript	Log_2 FC	Annotation	Process in which participate
**Hormone Signaling and Transcription Factors**				
T1	c47164_g1_i2	1,17	Abscisic acid receptor PYL8	ABA-mediated signalling
T2	c58196_g6_i1	2,49	Protein phosphatase 2C (PP2C)	ABA-mediated signalling
T3	c58196_g7_i2	3,73	Protein phosphatase 2C (PP2C)	ABA-mediated signalling
T4	c56735_g1_i8	5,74	Serine Threonine protein kinase	ABA-mediated signalling
T5	c50742_g2_i3	2,00	binding transcription factor activity ABA	ABA-mediated signalling
T6	c58895_g1_i3	-3,79	Carotenoid cleavage dioxygenase 8 (CCD8)	ABA synthesis
T7	c53673_g1_i1	2,03	9-cis-epoxycarotenoid dioxygenase (NCED)	ABA synthesis
T8	c100698_g1_i1	-9,60	Molybdenum cofactor sulfurase ABA3	ABA synthesis
T9	c54911_g1_i4	3,90	Glutamate N-acetyltransferase (EC:2.3.1.35)	Ornithine synthesis
T10	c49043_g1_i1	2,59	Ornithine decarboxylase (EC:4.1.1.17)	Ornithine synthesis
T11	c58141_g2_i2	3,59	ACC oxidase	ethylene synthesis
T12	c54745_g2_i1	1,59	CTR1	Ethylene-mediated signalling
T13	c58797_g5_i2	8,45	MES1	Ethylene-mediated signalling
T14	c49516_g1_i1	2,78	Small Auxin Up RNAs. SAUR32-like	SAUR family transcriptional regulator
T15	c53641_g1_i7	5,02	Transcription factor IAA10	Aux/IAA family transcriptional regulator
T16	c50693_g1_i2	-1,88	Indole-3-acetic acid-amido synthetase GH3	Auxin signalling
T17	c55410_g6_i1	-1,31	Serine/threonine-protein kinase CTR1	Ethylene signalling
T18	c54911_g1_i5	-1,86	Arabidopsis histidine kinase (AHK)	Cytokinin signalling
T19	c53387_g2_i1	-1,85	Two-component response regulator ARR6	Cytokinin signalling
T20	c59884_g1_i3	-6,81	Gibberellin-regulated protein 5 (GASA5)	GASA family protein regulated by gibberellin
T21	c46218_g1_i2	-3,37	Gibberellin-regulated protein 14 (GASA14)	GASA family protein regulated by gibberellin
T22	c49138_g1_i1	-3,01	Gibberellin 3 - oxidase 1	Gibberellin synthesis
T23	c55053_g1_i5	-2,83	2-Oxoglutaramate (2OG) and Fe(II)-dependent oxygenase superfamily protein	Xenobiotic degradation
T24	c46643_g3_i3	-4,86	Insensitive to jasmonate 3 (JAI3)	Jasmonic acid signaling
T25	c60049_g5_i1	-2,00	Arabidopsis 12-oxophytodienoate reductase 2 (OPR2)	Oxylipin synthesis
T26	c57485_g1_i1	-1,67	jasmonic acid synthase	Jasmonic acid signalling
T27	c46425_g1_i1	-3,72	Exordium like 7	Pro-brassinosteroid signaling
T28	c57040_g8_i1	-3,69	Sulfotransferase	Sulfonation of brassinosteroid precursors
T29	c5591_g1_i2	-4,85	Inflorescence receptor-like protein kinase (IMK2)	Leucine repeat receptor kinase
T30	c58835_g1_i6	-2,26	Cameliol C synthase 1 (CAMS1)	Cameliol synthesis
T31	c55023_g1_i5	-2,15	Sterol methyltransferase 2	Sterol biosynthesis
T32	c56884_g11_i8	9,52	mTOR-like, target protein of mammalian cell-like rapamycin	Regulation of cell growth, proliferation and death
T33	c56086_g1_i1	7,88	Transcription factor HSC1	Hsf family transcriptional regulator
T34	c55733_g1_i5	3,49	Transcription factor ATHB-12	Transcription regulator of the HD-Zip family
T35	c46935_g1_i1	2,41	Transcription factor ATHB-7	Transcription regulator of the HD-Zip family

T36	c52164_g1_i1	3,61	Transcription factor RD26	Transcriptional regulator of the NAC family
T37	c46885_g1_i1	2,87	Transcription factor sigE	Regulator of sigma transcription initiation
T38	c59823_g3_i1	2,24	Transcription factor *bZIP 56*	Transcriptional regulator of the bZIP family
T39	c51846_g2_i1	2,01	Transcription factor MYB59	Transcriptional regulator of the MYB family
T40	c59101_g3_i3	1,32	Transcription factor MYB73	Transcriptional regulator of the MYB family
T41	c53292_g1_i1	3,99	Transcription factor ERF10	ERF family transcription regulator
T42	c51584_g3_i1	3,22	Transcription factor EBP	ERF family transcription regulator
T43	c57392_g3_i3	3,07	Transcription factor ARR2	ARR family transcription factor
T44	c56273_g5_i1	2,15	Transcription factor WRKY4	Transcription regulator of the WRKY family
T45	c54019_g1_i2	2,97	Transcription factor DREB2C	Transcriptional regulator of the ERF/AP2 family
T46	c57837_g1_i16	2,12	Transcription factor GBF3	Transcriptional regulator of the G-box family
T47	c54696_g1_i4	2,28	Transcription factor ARF2	Auxin response transcriptional regulator
T48	c53641_g2_i1	1,69	Transcription factor IAA6	Aux/IAA family transcriptional regulator
T49	c50399_g1_i1	1,24	Transcription factor IAA29	Aux/IAA family transcriptional regulator
T50	c57040_g2_i3	5,26	Transcription factor HB2	Transcription regulator of the HB family
T51	c53177_g1_i8	2,80	Transcription factor RVE1	Transcriptional regulator of the MYB-like family
T52	c57725_g7_i2	2,63	Transcription factor HB40	Transcription regulator of the HB family
T53	c57364_g1_i1	1,53	Transcription factor WRKY33	Transcription regulator of the WRKY family
T54	c59785_g4_i8	2,71	Transcription factor ATAF1	Transcriptional regulator of the NAC family
T55	c59785_g4_i3	2,24	Transcription factor ARR1	ARR family transcription factor
T56	c59785_g4_i8	2,71	Transcription factor ARR11	ARR family transcription factor
T57	c44391_g1_i1	2,52	Transcription factor ZAT11	Factor de transcripcion de la familia C2H2
**Carbohydrate Metabolism**				
T58	c60260_g2_i14	5,52	1,4-alpha-glucan branching enzyme	Starch synthesis
T59	c40983_g1_i1	3,02	Alpha-amylase	Starch degradation
T60	c58521_g5_i7	4,46	Isoamylase	Starch degradation
T61	c55259_g1_i6	4,72	Sucrose synthase	Sucrose synthesis
T62	c55259_g1_i7	2,00	Sucrose synthase	Sucrose synthesis
T63	c53912_g1_i11	-2,26	Sucrose synthase	Sucrose synthesis
T64	c53912_g1_i11	-2,26	Sucrose synthase	Sucrose synthesis
T65	c53912_g1_i8	-2,44	Sucrose synthase	Sucrose synthesis
T66	c47708_g1_i1	-5,35	Beta-fructofuranosidase	Fructofuranoside hydrolysis
T67	c49833_g1_i1	2,66	Trehalose 6-phosphate phosphatase	Trehalose synthesis

T68	c49833_g1_i4	2,99	Trehalose 6-phosphate synthase	Trehalose synthesis
T69	c47636_g1_i1	3,43	Cellulose 1,4-beta-cellobiosidase	Cellulose degradation
T70	c49944_g1_i1	-5,27	Endoglucanase	Hydrolysis of cellulose β-1,4-glycosidic bonds
T71	c53655_g1_i3	9,56	Alpha-glucosidase	Alpha-glycosidic bond hydrolysis
T72	c58486_g4_i6	5,77	UDP-glucose 4-epimerase	Glucose/galactose epimerization
T73	c55610_g1_i4	3,16	Galactinol synthase	Galactinol synthesis
T74	c37638_g1_i1	-4,56	Raffinose synthase	Raffinose synthesis
T75	c56308_g4_i10	2,31	Alpha-galactosidase	Hydrolysis of melibiose to galactose
T76	c48740_g1_i5	-2,00	Hexokinase	Hexose phosphorylation
T77	c58333_g1_i6	-2,30	6-phosphofructokinase 1	Catalysis of the phosphorylation of fructose-6-phosphate
T78	c52064_g2_i5	-2,37	pyruvate kinase	Pyruvate production
T79	c57702_g1_i2	-2,74	pyruvate kinase	Pyruvate production
T80	c55561_g3_i1	-3,42	Pyruvate dehydrogenase component E1	Part of the pyruvate dehydrogenase complex
T81	c55279_g2_i2	-2,23	Pyruvate decarboxylase	Part of the pyruvate dehydrogenase complex
T82	c54512_g1_i1	-3,30	Pyruvate dehydrogenase component E2	Part of the pyruvate dehydrogenase complex
T83	c58019_g1_i10	-2,19	Dihydrolipoamide dehydrogenase	Part of the pyruvate dehydrogenase complex
**Amino acids and Proteins**				
T84	c59204_g6_i1	2,00	Ketolacid reductoisomerase	BCAA synthesis
T85	c54382_g1_i2	1,32	Acetolactate synth I/III small subunit	BCAA synthesis
T86	c59038_g7_i4	6,76	3-Isopropylmalate large subunit / (R)-2-methylmalate dehydratase	BCAA synthesis
T87	c46842_g1_i1	4,90	Similar to saccharopepsin	Protease activity
T88	c16690_g1_i1	3,11	cathepsin F	Protease activity
T89	c58480_g4_i6	2,53	Ubiquitin carboxy-terminal hydrolase 14	Protease activity
T90	c55994_g2_i1	8,51	Ubiquitin carboxy-terminal hydrolase 5	Protease activity
T91	c58965_g1_i2	8,82	puromycin sensitive aminopeptidase	Protease activity
T92	c59492_g12_i2	9,41	insulin-like	Protease activity
T93	c52024_g1_i1	4,70	Similar to deuterolysin	Protease activity
T94	c58616_g5_i2	2,05	FtsH cell division protease	Protease activity
T95	c58924_g3_i1	5,99	Endopeptidase STE24	Protease activity
T96	c52897_g1_i1	4,86	Trypsin-like	Protease activity
T97	c53434_g1_i1	4,80	Similar to subtilisin 8	Protease activity
T98	c52928_g1_i5	7,38	Serine carboxypeptidase-like Class II	Protease activity
T99	c58865_g1_i4	12,17	Alpha 6 subunit of the 20S proteasome	Protease activity
T100	c59894_g1_i1	8,58	threonine aspartase	Protease activity
T101	c57114_g1_i1	7,82	ribosomal genesis protein	GTP-binding protein
T102	c59440_g1_i16	7,30	L46 large subunit ribosomal protein	Participates in ribosomal assembly
T103	c56639_g2_i2	7,38	Small subunit ribosomal protein	Participates in ribosomal assembly
T104	c55243_g7_i7	2,03	U3 small nucleolar RNA-associated proteins 19	Cofactor of ribosomal biogenesis
T105	c59184_g7_i10	2,07	U3 small nucleolar RNA-associated proteins 25	Cofactor of ribosomal biogenesis

T106	c55752_g3_i14	3,34	adenylate kinase	Adenine nucleotide interconversion
T107	c55367_g1_i4	2,72	transportin 1 (TRN1)	Nuclear importer
T108	c55135_g3_i2	2,18	Eukaryotic translation initiation factor 1	Translation regulator
T109	c164642_g1_i1	6,99	Eukaryotic translation initiation factor 4A	Translation regulator
T110	c41469_g1_i1	7,40	Eukaryotic translation initiation factor 5A	Translation regulator
T111	c59164_g1_i18	7,63	Peptide chain releasing factor subunit 3	Translation regulator
T112	c54158_g1_i9	6,88	Aspartyl-tRNA synthetase	Transfer RNA loading
T113	c56681_g2_i1	9,08	Valil-tRNA synthetase	Transfer RNA loading
T114	c9595_g1_i1	6,40	Late embryogenesis proteins(LEA)	Protection against dehydration
T115	c47143_g1_i1	2,01	Late embryogenesis proteins(LEA)	Protection against dehydration
T116	c49408_g1_i3	9,19	Late embryogenesis proteins(LEA)	Protection against dehydration
T117	c43538_g1_i1	3,50	Late embryogenesis proteins(LEA)	Protection against dehydration
**Overexpressed transcripts for which proteins in the interactome were annotated**				
T118	c50221_g1_i1	4,08	GTP-binding elongation factor	Translation regulator
T119	c41469_g1_i1	7,40	Eukaryotic translation initiation factor 5A-3	Translation regulator
T120	c58899_g2_i2	2,73	Gen 24 associated with senescence	Regulation of senescence
T121	c56779_g1_i5	1,37	18S ribosomal protein 40S	Structural constituent of ribosomes
T122	c50992_g1_i1	3,08	polyubiquitin 4	Proteasome recognition signal
T123	c53038_g3_i1	2,47	polyubiquitin 8	Proteasome recognition signal
T124	c34438_g1_i1	4,19	Ubiquitin-conjugating enzyme E2 11	Ubiquitin-mediated proteolysis
T125	c47011_g1_i1	7,06	S phase kinase-associated protein 1	Cell cycle control
T126	45226_g1_i1	4,92	Pectin methyl esterase 58	Cell wall metabolism
T127	c57790_g1_i1	3,10	actin 1	Cytoskeletal constituent
T128	c47244_g1_i3	3,66	Cysteine peptidase xylem 2	Cell wall metabolism
T129	c57966_g6_i1	7,66	Probable xyloglucan endotransglucosylase	Cell wall metabolism
T130	c48780_g1_i2	3,21	peroxidases	Hydrogen peroxide catabolism
T131	c52403_g1_i4	3,48	Subtilisin-like endopeptidase family protein	Protease activity
T132	c42692_g1_i1	4,80	Heat shock protein 90	Hsf family transcriptional regulator
T133	c44151_g1_i1	2,66	Heat shock protein 70	Hsf family transcriptional regulator
T134	c58788_g3_i1	2,14	Annexin 8	Modulador de actividad fosfolipásica
T135	c53523_g2_i1	3,48	Binding protein causing deficiency in dependent gravitropism	Protease activity
T136	c120137_g1_i1	2,45	U-box domain protein 18	Ubiquitin transferase
T137	c55610_g2_i1	3,63	Galactinol synthase 2	Galactinol synthesis
T138	c46526_g3_i1	7,60	Calcium-dependent protein kinase 27	Calcium-dependent protein kinase
T139	c50226_g3_i1	2,04	Monodehydroascorbate reductase 3	Dehydroascorbate reduction
T140	c41284_g1_i1	3,87	Cu-Zn 2 superoxide dismutase (CSD2)	Superoxide radical catabolism
T141	c21935_g1_i1	2,68	Dehydration response peptidase cysteine 21B	Protease activity
T142	c120812_g1_i1	6,55	Similar to betaine glyceraldehyde dehydrogenase	Glycinbetaine synthesis
T143	c40024_g3_i1	6,99	Sugar-binding protein 13	Hexose/H+ symport
T144	c41462_g1_i1	3,89	Glycine-rich RNA-binding protein 4	Stress response
T145	c57036_g3_i1	3,74	Radical-induced cell death protein 1	Regulation of oxidative stress
T146	c13289_g2_i1	4,47	Respiratory bud oxidase homolog F protein	Superoxide generation
T147	c18843_g1_i1	7,40	GroES-like alcohol dehydrogenases	Alcohol oxidation

T148	c60145_g2_i1	3,70	GroES-like alcohol dehydrogenases	Alcohol oxidation
T149	c24396_g1_i1	4,87	GroES-like alcohol dehydrogenases	Alcohol oxidation
T150	c60145_g1_i1	3,63	GroES-like alcohol dehydrogenases	Alcohol oxidation
T151	C53673_g2_i1	1,07	9-cis epoxycarotenoid dioxygenase (NCED)	ABA synthesis

Supplementary Table S6. Primers used for transcript level quantification by real-time qPCR.

**Gene**	**Primer name**	**Sequences (5'→3')**	**Amplicon length (bp)**	**Efficiency factor**	**Regression coefficient (R^2 value)**
1,4-alpha-glucan branching enzyme *(GBE1)*	BRAN	F: TTGCCCTTGCTCAGATTG<br>R: TCACTCACCCACACCAAAC	181	1.96	0.999
Galactinol synthase (GolS)	GOL	F: ACTATTGTGCTGCGGGTTC<br>R: GGTGCGGTAACGGAATAAAC	196	1.98	0.986
1-aminocyclopropane-1-carboxylate oxidase (ACC)	ACC	F: GGACTTGCTATGTGAGAACC<br>R: CTGGAAGAGGAGGATGATG	178	1.94	0.998
Trehalose phosphate synthase (TPS)	TREH	F: GGTCTGGTGTTATGAGGATG<br>R: GCAAGGTAGAAAGTAGGCG	185	1.94	0.997
Alternative oxidase (AOX)	AOX	F: GGTGGTGGGGATGATAAG<br>R: CAAGAAAGTCACAGGGGC	168	1.96	0.997
Polyphenol oxidase (PPO)	PPO	F: TTACCAACCAGTGCCTATTC<br>R: CCTCTTCATCTTCCTTTTCC	188	1.95	0.994
Raffinose synthase (RafS)	RAF	F: CCCTCCAACAAGAAACCAG<br>R: GAAGTCAGTCTCAGCATCGG	183	1.95	0.995
RS-1 protein (RSI-1)	RSI	F: GTCTCGCTCTCTCTCTTTTTTC<br>R: CGGATTTTACACTACTGCCTC	154	1.96	0.984
Uncharacterized transcript (c58016_g2_i2)	UNTR	F: GTTTCTGGGTGGTGGTTTC<br>R: GCTGGTTGATCCTTCAGTTG	161	1.97	0.998
RNA polymerase-associated protein rtf1 (RTF1)	RTF	F: AGAGGGCTGCTCTGAAATGC<br>R: TGAACTCGAACCTCGTCGTC	85	1.95	0.994
